# Computational paradigms for antimicrobial resistance prediction: integrating multi-omics, structural modeling, and foundation artificial intelligence systems

**DOI:** 10.1093/bib/bbag219

**Published:** 2026-05-11

**Authors:** Elias Hossain, Niloofar Yousefi

**Affiliations:** Department of Industrial Engineering and Management Systems, University of Central Florida, 12800 Pegasus Dr, Orlando, FL 32816, USA; Department of Industrial Engineering and Management Systems, University of Central Florida, 12800 Pegasus Dr, Orlando, FL 32816, USA

**Keywords:** antimicrobial resistance, Salmonella enterica, multi-omics, whole-genome sequencing, antimicrobial resistance prediction, transformer-based models, large language models, One Health

## Abstract

Antimicrobial resistance (AMR) poses an escalating threat to global health, as multidrug-resistant pathogens undermine therapeutic efficacy and surveillance systems. Although whole-genome sequencing and phenotypic drug susceptibility testing have strengthened resistome profiling, translating multi-omics data into reliable, clinically deployable intelligence remains computationally fragmented. Following PRISMA 2020 guidelines, we systematically reviewed 156 records published between 2016 and 2025, of which 93 studies were included in the final synthesis. We organize AMR modeling into three methodological strata: (i) classical and interpretable machine-learning frameworks, (ii) structural and deep genomic architectures, and (iii) transformer-based and applied large language model systems that integrate genomic, clinical, and epidemiological signals. Across studies, we identify four converging integrative directions: embedding-level multimodal fusion, knowledge-graph-guided causal reasoning, evolutionary and temporal forecasting, and agentic artificial intelligence systems enabling autonomous, evidence-grounded workflows. Comparative analysis reveals substantial heterogeneity in dataset scale, frequent reliance on internal validation, limited assessment of cross-site robustness, and vulnerability to distribution shift, particularly for minority resistance phenotypes. We argue that future AMR intelligence must integrate uncertainty-aware modeling, standardized validation protocols, and FAIR-compliant infrastructures to transition from static genomic classification toward interpretable, temporally adaptive, and clinically actionable decision systems within One Health surveillance ecosystems.

## Introduction

Antimicrobial resistance (AMR) is widely recognized as one of the most pressing challenges to global public health [1, 2], threatening to undermine decades of progress in infectious disease prevention and clinical treatment. The rapid spread of resistant bacteria has rendered many first-line and last-resort antibiotics less effective [3, 4], thereby increasing morbidity, mortality, and healthcare costs worldwide [5–7]. In practice, the AMR burden manifests through a connected workflow: surveillance detects emerging resistance and its spread, diagnostics confirm susceptibility, clinicians select therapy under time pressure, and stewardship programs guide antibiotic use to slow resistance amplification [8–11].

Among bacterial pathogens, *Salmonella enterica* represents a particularly critical concern because of its pervasive presence in food systems, its zoonotic transmission pathways, and the growing prevalence of multidrug-resistant (MDR) strains across human, animal, and environmental reservoirs [12–14]. These characteristics position *Salmonella* as both a sentinel organism and a representative model for studying the emergence, evolution, and dissemination of AMR across One Health settings.

A key enabler of modern surveillance is whole-genome sequencing (WGS), which enables high-resolution tracking of lineages, resistance determinants, and dissemination routes across reservoirs [15, 16]. At the clinical interface, phenotypic drug susceptibility testing (pDST) [17, 18] remains central to confirming resistance. However, translating WGS outputs into actionable decisions and scaling timely confirmation through pDST remain challenging in practice because of heterogeneity in sampling and metadata, limitations in sequencing and analysis capacity, turnaround time, laboratory workload, cost, and inconsistent standardization [19–21]. These bottlenecks can delay effective therapy and complicate outbreak response, thereby motivating computational approaches that anticipate resistance earlier from genomic and contextual signals to support precision treatment while explicitly communicating uncertainty and failure modes.

Beyond methodological performance, AMR modeling plays a central role in modern microbial surveillance and precision-treatment strategies. In contemporary health systems, WGS-enabled surveillance and digital antibiograms increasingly function as integrated infrastructures for tracking resistance emergence, detecting outbreak signals, and guiding empiric and targeted antimicrobial therapy. Predictive models are therefore not isolated analytical tools; rather, they operate as components within surveillance ecosystems that inform antibiotic selection, escalation or de-escalation decisions, and regional resistance monitoring under a One Health framework. As resistance determinants evolve across time, geography, and host populations, clinically relevant AMR systems must support real-time interpretability, uncertainty awareness, and deployment under heterogeneous laboratory and infrastructure conditions. Framing AMR prediction within this broader surveillance and precision-treatment context is essential for evaluating translational impact rather than reporting performance metrics in isolation [8, 10, 15, 16].

At the molecular level, AMR arises through a combination of spontaneous genetic mutations and horizontal gene transfer mechanisms that enable bacterial populations to adapt rapidly under selective pressure [22, 23]. Resistance phenotypes do not arise solely from single genetic mutations; they may also reflect collective and context-dependent mechanisms, including biofilm-mediated protection and antibiotic inactivation within microbial communities [24]. Taken together, the difficulty of converting WGS signals into reliable decision support and the delays introduced by pDST highlight a central modeling limitation: marker-centric surveillance and prediction approaches that focus on single mutations or a narrow set of resistance genes may miss context-dependent resistance driven by community interactions and physiological state. As a result, predictive models that rely primarily on single-omics features or fixed genetic markers may underperform when resistance emerges from collective protection, altered growth dynamics, or local antibiotic inactivation, thereby motivating integrative computational frameworks that capture higher-order, interaction-driven dependencies.

In response to these challenges, computational modeling has become increasingly central to AMR research. Early approaches primarily employed classical machine-learning methods using handcrafted genomic features such as single nucleotide polymorphisms [25–27], *k*-mers [28–30], and gene presence–absence matrices [31]. While these models offered interpretability and computational efficiency, their ability to represent complex dependencies and context sensitivity remained limited. Subsequent advances in deep learning introduced convolutional, graph-based, and sequence-aware architectures that improved representational power and enabled more expressive modeling of resistance determinants [32–34]. More recently, transformer-based models and large language models (LLMs) have further expanded this paradigm by supporting long-range dependency modeling and scalable integration of genomic, clinical, and textual data sources [35–37]. As model capacity increases, concerns regarding robustness under distribution shift, interpretability, calibration, reproducibility, and deployment readiness become more acute in high-stakes clinical and public-health settings.

Concurrently, the growing availability of high-throughput sequencing and multi-omics measurements (e.g. transcriptomics, proteomics, metabolomics, and dual RNA-seq) has reshaped the analytical foundation of predictive microbiology by revealing regulatory and physiological dimensions of resistance beyond static genetic determinants. Yet, integrating heterogeneous modalities remains difficult because of data harmonization issues, incomplete pairing across modalities, limited matched cohort sizes, batch effects, platform variability, and confounding introduced by population structure and sampling bias. These challenges frequently emerge during deployment as brittleness across sites and time, as well as failures to calibrate confidence for decision-making. LLM-enabled systems add new capabilities for evidence synthesis and workflow orchestration, but they also introduce additional requirements for grounding to verifiable sources, controlling unfaithful generation, handling sensitive clinical metadata, and meeting real-time cost and scalability constraints.

This review provides a focused synthesis of computational paradigms for AMR prediction, with an emphasis on *Salmonella* and related foodborne pathogens. We examine the evolution of modeling approaches from classical machine learning to deep genomic architectures, transformer-based models, and emerging agentic artificial intelligence (AI) systems. Beyond summarizing methods, we organize the literature around the practical bottlenecks that determine real-world impact, including WGS-to-action translation, pDST delays, multimodal data integration, and deployment constraints, while highlighting design choices that improve interpretability, uncertainty awareness, and operational fit. Specifically, the contributions of this review are:

- A structured taxonomy of AMR prediction approaches (classical ML, deep genomic models, and transformer- and LLM-based systems), clarifying what each paradigm captures, assumes, and fails to capture.
- A synthesis of integrative frameworks that bridge genomic signals to actionable intelligence, including multimodal fusion strategies, knowledge-graph (KG) and causal reasoning, evolutionary and temporal forecasting, and agentic workflows.
- A consolidated analysis of persistent barriers, including data heterogeneity, limited multimodal pairing, robustness under shift, calibration and uncertainty reporting, interpretability, and deployment cost, together with the evaluation practices needed for clinical and surveillance adoption.
- A roadmap connecting computational advances to precision-treatment and stewardship objectives, with implications for real-time outbreak monitoring in One Health settings.

## Antimicrobial resistance and multidrug-resistant *Salmonella*

### Genetic basis of AMR and the emergence of MDR *Salmonella*

AMR arises from the ability of bacterial genomes to adapt under selective pressure through well-characterized genetic mechanisms. Resistance emerges via **spontaneous chromosomal mutations** or, more prominently, through **horizontal gene transfer**, which enables the rapid acquisition of resistance determinants across bacterial populations. Mobile genetic elements, including plasmids, integrons, and transposons, play a central role by facilitating the dissemination of resistance genes across species boundaries and ecological niches.

Within the genus *Salmonella*, horizontally acquired resistance has contributed to the emergence of **MDR non-typhoidal strains**, thereby complicating both clinical management and epidemiological control. MDR *Salmonella enterica* isolates frequently exhibit co-resistance to multiple antibiotic classes, including ampicillin, chloramphenicol, and trimethoprim, with additional resistance to fluoroquinolones and third-generation cephalosporins reported in specific lineages. These phenotypes are often mediated by plasmid-borne genes such as *bla_TEM_, catA1*, and *dfrA*, which collectively undermine standard therapeutic regimens in both human and veterinary settings [38].

**Figure 1:**
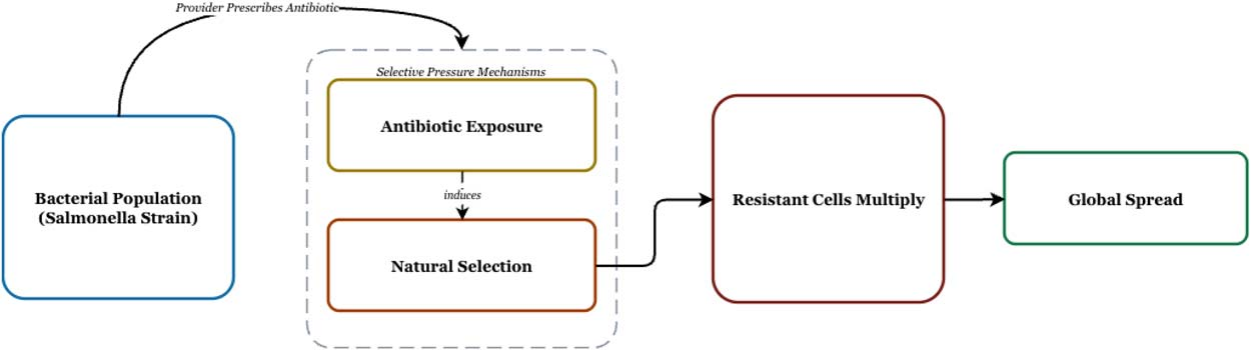
**Mechanisms of AMR emergence and dissemination**. The schematic illustrates how antibiotic exposure and natural selection promote resistant Salmonella enterica variants, enabling resistant cells to multiply and spread across human, animal, food, and environmental reservoirs within a One Health transmission framework [41].

Sustained antimicrobial exposure in healthcare, agriculture, and food-production systems imposes strong selective pressure: susceptible subpopulations are eliminated, whereas resistant clones persist and expand. Over time, these lineages disseminate through interconnected food, animal, and environmental reservoirs, forming a reinforcing cycle of resistance amplification (Fig. 1). In the USA, epidemiological surveillance has documented a marked increase in the prevalence of the emerging MDR serotype *S. enterica* 4,[5],12:i:-, particularly among swine- and human-associated isolates in the Midwest [39]. This lineage illustrates the interaction among antimicrobial use in livestock, zoonotic transmission pathways, and plasmid-mediated gene flow.

From a **One Health** perspective [40], the persistence and dissemination of MDR *Salmonella* underscore the need for coordinated surveillance and mitigation strategies spanning human, animal, and environmental health domains. Effective responses require integrated antimicrobial stewardship, as well as vaccination and biosecurity interventions in food-animal production, together with large-scale genomic monitoring of resistance determinants to track emerging MDR lineages and inform evidence-based policy.

### WGS-based resistome profiling in MDR *Salmonella*

WGS has become a central component of AMR surveillance because it enables direct characterization of the genetic determinants underlying observed resistance phenotypes. In MDR *Salmonella enterica*, WGS provides nucleotide-level resolution of both horizontally acquired resistance genes and chromosomal mutations that contribute to reduced antibiotic susceptibility. This dual capability is essential because MDR phenotypes in *Salmonella* often arise from the combined presence of plasmid-borne determinants and lineage-specific point mutations that alter antimicrobial targets.

Resistome profiling using WGS follows a structured analytical workflow. After quality control, sequencing reads may be analyzed directly or assembled into contigs or complete genomes. Annotation pipelines then screen these sequences against curated AMR gene databases. Gene-centric approaches identify acquired resistance genes through sequence homology or model-based inference, whereas mutation-centric approaches detect nucleotide substitutions associated with resistance in chromosomal loci. In MDR *Salmonella*, resistance to beta-lactams, aminoglycosides, sulfonamides, and tetracyclines is frequently mediated by mobile genetic elements, whereas reduced susceptibility to fluoroquinolones commonly involves chromosomal mutations. Consequently, comprehensive resistome profiling typically requires support for both gene-level and mutation-level detection.

However, translation from WGS data to resistance interpretation is not uniform across computational resources. As summarized in Tables 1 and 2, annotation tools differ substantially in their supported input types (raw reads, assembled genomes, or metagenomes) and in the forms of resistance evidence they report (acquired genes versus chromosomal mutations). Some resources emphasize rapid gene screening from assemblies, whereas others support metagenomic abundance profiling or mutation-focused interpretation. These differences can influence downstream resistance inference, particularly when comparing results across laboratories or surveillance networks.

**Table 1:** **ARG annotation resources and tools used in WGS/metagenomic AMR workflows (capability matrix, Part I).** This table compares widely used tools and databases by (i) **supported input type** and (ii) **the type of resistance evidence** produced by each tool.

Tool/resource	Reads	Asm	Meta	Mut	Genes	Summary
ResFinder [43]	✓	✓	$\times$	✓	✓	Clinical-oriented calling of acquired genes and selected chromosomal resistance mutations.
ARGs-OAP [44]	✓	$\times$	✓	$\times$	✓	Metagenomic ARG profiling; emphasizes abundance/coverage rather than phenotype-level calls.
ARDB (DB) [45]	$\times$	$\times$	$\times$	$\times$	✓	Legacy ARG database; useful historically but not maintained.
CARD (DB + models) [46]	✓	✓	✓	✓	✓	Curated ontology-backed resource supporting gene and variant interpretation across data types.
ARGO (DB) [47]	$\times$	$\times$	$\times$	$\times$	✓	Focused database (e.g. $\beta$-lactamase-rich); narrower coverage than general catalogs.
DeepARG [48]	✓	✓	✓	$\times$	✓	ML-based ARG prediction; improves sensitivity but requires careful thresholding.
GROOT [49]	✓	$\times$	✓	$\times$	✓	Fast resistome profiling for metagenomes; reporting is typically summary-level.
KmerResistance [50]	✓	$\times$	$\times$	$\times$	✓	Very fast WGS screening using $k$-mers; may miss divergent alleles outside the index.
AMRFinderPlus [51]	$\times$	✓	$\times$	✓	✓	Curated calling for acquired/intrinsic genes; mutation support is schema-dependent.
RAST/PATRIC [52]	$\times$	✓	$\times$	$\times$	✓	General genome annotation ecosystems with AMR modules; AMR specificity varies by setup.
ARG-ANNOT (DB) [53]	$\times$	✓	$\times$	$\times$	✓	DB-driven ARG detection from genomes; phenotype mapping depends on DB maintenance.
sraX [54]	$\times$	✓	$\times$	$\times$	✓	Automated resistome reporting for bacterial genomes; geared to rapid summary outputs.
Abricate [55]	$\times$	✓	$\times$	$\times$	✓	Batch contig scans against multiple DBs; typically does not capture point-mutation resistance.

*Note:*  **Reads**, raw short-read/long-read sequencing input; **Asm**, assembled contigs/genomes; **Meta**, metagenomic samples; **Mut**, chromosomal resistance mutations/variants; **Genes**, acquired/intrinsic ARG gene detection. Symbols: ✓ supported / typical use; $\times$ not supported or not a primary use case

**Table 2:** **ARG annotation resources and tools used in WGS/metagenomic AMR workflows (capability matrix, Part II).** Continuation of the capability-oriented comparison across additional tools and resources.

Tool/resource	Reads	Asm	Meta	Mut	Genes	Summary
SEAR [56]	✓	$\times$	✓	$\times$	✓	Detection of horizontally acquired ARGs from raw metagenomic reads (web-based workflow).
SSTAR [57]	$\times$	✓	$\times$	$\times$	✓	Standalone WGS ARG detection; flags truncated candidate ARGs.
MEGARes (DB) [58]	$\times$	$\times$	✓	$\times$	✓	Catalog including ARGs plus biocide/heavy-metal resistance genes; widely used in resistome studies.
MARA (DB) [59]	$\times$	$\times$	$\times$	$\times$	✓	Database focused on Gram-negative mobile ARGs; useful for plasmid/mobile element analyses.
FARME DB [60]	$\times$	$\times$	✓	$\times$	✓	Emphasizes mobile elements and flanking genes in metagenomic contexts.
ARGA (DB) [61]	$\times$	$\times$	$\times$	$\times$	✓	Resource emphasizing ARG primer design and assay development use cases.
ARGminer [62]	$\times$	$\times$	$\times$	$\times$	✓	Crowdsourced ARG curation platform supporting community-driven refinement of annotations.
ResistoMap [63]	$\times$	$\times$	✓	$\times$	✓	Interactive visualization platform for ARG patterns (e.g. gut microbiome profiles).
ARGs-OSP [64]	$\times$	$\times$	✓	$\times$	✓	Search platform over large WGS/metagenome collections for ARG discovery and comparison.
LRE-Finder [65]	$\times$	✓	$\times$	✓	$\times$	Targeted mutation analysis (23S rRNA) for linezolid resistance from WGS.
MvirDB [66]	$\times$	$\times$	$\times$	$\times$	✓	Database combining resistance genes with virulence/toxin annotations.
MUBII-TB-DB [67]	$\times$	$\times$	$\times$	✓	$\times$	Mutation-focused resistance resource for *Mycobacterium tuberculosis* (specialized scope).

*Note:*  **Reads**, raw sequencing input; **Asm**, assembled genomes/contigs; **Meta**, metagenomic input; **Mut**, resistance mutations/variants; **Genes**, ARG gene detection. Symbols: ✓ supported/typical use; $\times$ not supported or not a primary use case.

Beyond gene detection, WGS supports contextual analysis of resistance determinants within their genomic environment. Plasmid reconstruction and co-localization analyses help determine whether multiple resistance genes are physically linked, which has important implications for co-selection and horizontal dissemination. Integrating resistome data with phylogenetic analysis further supports outbreak investigation and lineage tracking, particularly for emerging MDR *Salmonella* serotypes.

Taken together, the comparative landscape summarized in Tables 1 and 2 highlights that resistome interpretation is inherently dependent on the computational layer applied to WGS data. For MDR *Salmonella*, in which resistance phenotypes often reflect interacting genetic mechanisms, consistent and well-characterized annotation strategies are essential for reliable surveillance, antimicrobial stewardship, and evidence-based decision-making. While the original review provides a descriptive listing of resistance-gene resources [42], we restructure the comparison into a capability-oriented matrix (Tables 1 and 2) to facilitate methodological assessment across WGS workflows.

## Methodology

### Literature search and selection strategy

We conducted a comprehensive literature review across multiple major electronic and preprint databases covering the years 2016 to 2025. The objective was to identify all relevant studies investigating multi-omics data integration and LLM applications in bacterial research, particularly in relation to *Salmonella*. Our review adhered to the PRISMA 2020 guidelines to ensure transparency and reproducibility throughout the study-selection process. The databases searched included PubMed, NCBI, bioRxiv, Elsevier, IEEE Xplore, Nature Portfolio, Oxford University Press, PLOS One, and Springer. These sources were selected because of their extensive coverage of high-quality research in computational biology and bioinformatics. To ensure a thorough and accurate search, we developed a systematic keyword strategy that combined controlled vocabulary terms, such as Medical Subject Headings (MeSH), with free-text expressions. In addition, Boolean operators were used to connect related concepts, thereby enabling the retrieval of both established studies and emerging trends in omics-based and AI-driven modeling. Table 3 presents the final keyword combinations and Boolean framework used in the search. We also employed wildcard operators (e.g. “LLMs*” and “multiomics*”) to capture variations in key terms and to accommodate the evolving vocabulary of the field. To further enhance coverage, we manually examined the reference lists of highly cited articles to identify relevant studies that may not have been captured by the initial keyword search.

**Table 3:** Keyword schema and Boolean logic used to conduct the systematic literature search. This table summarizes the search strategy designed to identify studies examining the intersection of computational modeling, multi-omics data integration, and AMR prediction in bacterial systems, including *Salmonella*. The Boolean operators linking each conceptual category were intended to capture both established methodologies and emerging research in AI-driven genomics. The strategy included both controlled vocabulary terms and free-text expressions. By integrating four conceptual domains—omics, computational models, biological context, and application focus—the final search string provided a retrieval framework that was both comprehensive and precise across databases from multiple disciplines.

Concept	Keywords and Boolean operators
**Omics Domain**	”genomics” OR ”metagenomics” OR ”epigenomics” OR ”transcriptomics” OR ”proteomics” OR ”metabolomics” OR ”multi-omics” OR ”multiomics” OR ”resistome” OR ”pan-genome”
**Computational Model**	”large language model” OR ”LLM” OR ”transformer” OR ”foundation model” OR ”representation learning” OR ”deep learning” OR ”neural network” OR ”AI” OR ”machine learning” OR ”BERT” OR ”BioBERT” OR ”BioGPT” OR ”DNABERT”
**Biological Context**	”Salmonella” OR ”Escherichia coli” OR ”pathogen” OR ”bacterial genome” OR ”antimicrobial resistance” OR ”AMR gene” OR ”foodborne pathogen” OR ”microbial surveillance” OR ”infection prediction”
**Data and Application Focus**	”omics integration” OR ”gene prediction” OR ”functional annotation” OR ”drug resistance” OR ”phenotype classification” OR ”host–pathogen interaction” OR ”disease modeling”
**Final Search String**	(Omics Domain) AND (Computational Model) AND (Biological Context) AND (Data and Application Focus)

### Selection criteria

To ensure that all selected studies were relevant to the scope of this review, the inclusion criteria were carefully defined to emphasize integrative and computational methodologies in *Salmonella* research. We considered articles that used multi-omics datasets, including genomic, transcriptomic, proteomic, or metabolomic data, as well as studies related to bacterial pathogens more broadly. We also included studies that applied deep learning, machine learning, LLMs, or other state-of-the-art approaches to the development of predictive models for *Salmonella* resistance, with particular emphasis on those incorporating multi-omics data. For study selection, we considered publications from peer-reviewed journals, conference proceedings, and high-quality preprint repositories published between 2016 and 2025. Only studies published in English and providing sufficient methodological detail to allow independent evaluation were retained.

To maintain scientific rigor and focus, we also applied exclusion criteria. Specifically, we excluded editorials, studies lacking empirical evaluation or methodological contribution, and publications identified as predatory. We further excluded studies that did not address omics integration, machine learning, or microbial AMR prediction. In addition, publications with inaccessible full texts, incomplete records, or duplicate entries were removed during screening. Rule-based or expert-driven systems that did not incorporate data-driven inference or model interpretability were generally not prioritized unless they offered distinctive methodological or conceptual contributions of clear relevance to computational biology.

### Search output and screening process

**Figure 2:**
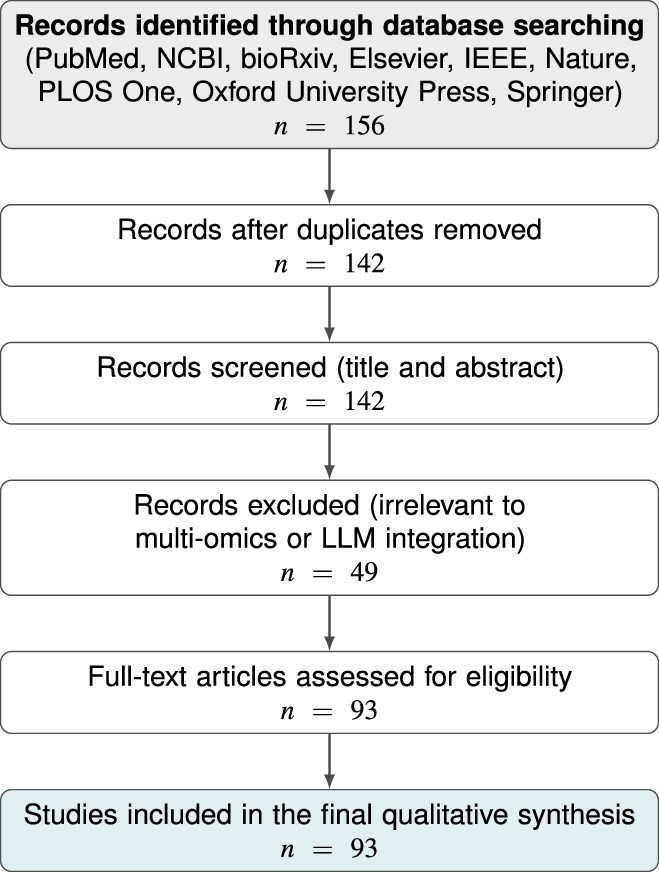
PRISMA flow diagram illustrating the literature screening and study selection process. The diagram shows that 156 records were initially identified through database searching. After removing 14 duplicate records, 142 studies were screened by title and abstract, 49 were excluded as irrelevant, and 93 full-text articles were assessed for eligibility. The final qualitative synthesis included 93 studies.

Figure 2 presents the PRISMA flow diagram summarizing the literature retrieval and screening workflow. In total, 156 records were initially identified across all databases. After the removal of 14 duplicate entries, 142 unique studies were screened based on title and abstract. Of these, 49 were excluded because they were not relevant to multi-omics analysis or LLM-based modeling. The remaining 93 studies underwent full-text assessment for eligibility and methodological quality, and all 93 were included in the final qualitative synthesis. For each included study, we further categorized the omics data type, computational paradigm (classical machine learning, deep learning, or transformer-based), integration level (data-, feature-, or decision-level), and AMR-related outcome metrics.

### Quality assessment and data extraction

To mitigate bias and ensure methodological consistency, each eligible study was evaluated using a structured quality-appraisal process. The assessment focused on several key dimensions, including the reproducibility of model design and training procedures, the accessibility and completeness of the underlying datasets, the transparency of the reported evaluation metrics, and the clarity of feature-engineering or data-integration descriptions. In addition, we used a five-point internal scale to assess each publication, with higher scores indicating greater methodological transparency, robustness, and reproducibility. This structured approach provided a consistent basis for comparing studies that employed diverse analytical paradigms, ranging from traditional machine-learning classifiers to transformer-based LLMs.

Following quality assessment, relevant information was extracted to support comparative synthesis. The extracted information included bibliographic details, computational or model design characteristics, evaluation metrics, interpretability methods, and reported biological outcomes related to antibiotic resistance prediction or functional genomics. To facilitate cross-study comparison and pattern identification, all extracted information was organized and analyzed in a consistent manner. The final dataset was then used to identify gaps in the existing literature, key data-integration strategies, and common modeling approaches.

## Survey of existing AMR modeling approaches

**Figure 3:**
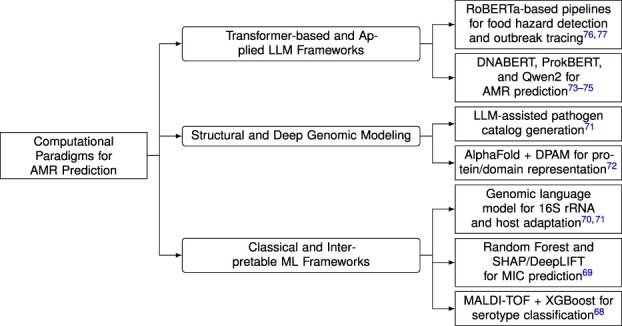
Taxonomy of computational paradigms for AMR prediction. The hierarchy consolidates existing computational strategies into three methodological classes: (i) **Classical and Interpretable ML Frameworks**, which include feature-engineered and hybrid interpretable models; (ii) **Structural and Deep Genomic Modeling**, which integrates protein- and domain-level representations; and (iii) **Transformer-based and Applied LLM Frameworks**, encompassing foundation-level genomic models and public-health-oriented pipelines. Each node represents a key methodological direction and its representative studies.

The taxonomy illustrated in Fig. 3 provides a comprehensive overview of the evolution of computational methods for AMR prediction. It visually represents the transition from early feature-based learning models to advanced foundation-level architectures capable of interpreting complex genomic patterns. This taxonomy captures both the historical progression and the methodological diversity of the field, demonstrating how advances in data integration, model interpretability, and large-scale computation have shaped contemporary AMR research. In general, the taxonomy divides AMR modeling studies into three primary methodological categories. The first corresponds to the early phase of computational research, which was dominated by classical and interpretable machine learning techniques. The second reflects the transition to deep genomic and structure-informed modeling, highlighting advances in multi-omics and protein-level representation learning. The third emphasizes the emergence of transformer-based and applied LLM frameworks that integrate genomic, textual, and epidemiological data into unified predictive systems. Together, these three categories provide the conceptual foundation for the discussion presented in this section, which reviews representative studies, modeling strategies, and emerging trends.

### Computational paradigms in AMR modeling

Recent research reflects three primary methodological categories corresponding to distinct stages in the evolution of AMR modeling. The first category, *classical and interpretable machine learning frameworks*, includes approaches based on interpretable algorithms such as Random Forest and XGBoost, manually engineered genomic features, and hybrid models that incorporate explainability methods such as SHAP and DeepLIFT. The second category, *structural and deep genomic modeling*, seeks to integrate protein structure, domain-level features, and deep biological representations using methods such as AlphaFold and domain-aware attention mechanisms. The third category, *transformer-based and applied LLM frameworks*, includes advanced models such as DNABERT, ProkBERT, and Qwen2, which capture contextual dependencies across genomic and epidemiological data and enable large-scale reasoning for AMR prediction. Each methodological category reflects a different stage in the development of AMR research, ranging from early statistical and interpretable models to recent transformer-based systems. A more detailed review of these categories is provided in the following sections, with a focus on modeling techniques, representative studies, and the distinctive contributions of each category to the broader field of AMR prediction.

#### Classical and interpretable machine learning frameworks

Early computational efforts in AMR prediction primarily relied on feature engineering and conventional classifiers, including Random Forest and XGBoost. These models used sequence-derived representations such as *k*-mers, genomic signatures, and MALDI-TOF embeddings to classify serotypes, infer microbiome-level risk, and identify host-adaptation signatures [68, 70, 78]. Despite their efficiency and relatively low computational overhead, they primarily captured local sequence statistics and were limited in their ability to represent higher-order genomic context or long-range dependencies. To improve interpretability, subsequent studies developed hybrid modeling frameworks that combined traditional machine learning algorithms with neural attribution methods, including DeepLIFT and SHAP. These approaches provided deeper insight into model decisions by identifying the contributions of specific genomic loci, protein features, or regulatory motifs, thereby supporting tasks such as phenotype classification and minimum inhibitory concentration (MIC) prediction [69, 71, 79]. This methodological refinement helped bridge biological insight and statistical learning, enabling researchers to trace the effects of specific genomic variations on AMR outcomes.

However, despite their success in classification and feature attribution, classical machine learning approaches remained limited in their ability to capture hierarchical genomic dependencies and contextual relationships across multi-omics data. Their scalability was also constrained when applied to large and heterogeneous microbial populations because of their reliance on handcrafted features and relatively isolated datasets. Nevertheless, these models established a crucial foundation for AMR prediction research, which was later extended through the development of deep learning and transformer-based approaches.

**Key insight: bridging interpretability and complexity**

The early phase of AMR modeling highlights a persistent trade-off between interpretability and representational depth. Classical models remain transparent and reproducible, but they struggle to generalize across complex genomic contexts. This ongoing challenge underscores the need to advance interpretable frameworks toward scalable, context-aware systems while preserving clarity and biological relevance.

#### Structural and deep genomic modeling

As AMR research progressed, predictive modeling pipelines increasingly incorporated structural biology and protein-domain knowledge. Rather than relying exclusively on nucleotide sequences, researchers began integrating protein structure and domain-level information to better understand how molecular conformation shapes resistance mechanisms. These approaches combined domain-adaptive attention networks, such as DPAM, with structure-aware representations, including AlphaFold-derived embeddings, to support structure-informed resistance inference [72]. By modeling 3D conformations and spatial interactions, such frameworks enabled AMR prediction systems to move beyond surface-level correlations toward mechanistic explanations grounded in protein function.

In parallel, large-scale efforts using LLM-assisted literature mining and pathogen catalog curation contributed to the development of functional annotation frameworks that link sequence-level signals with protein conformation [71]. These initiatives incorporated structured and evolutionary annotations into genomic knowledge bases, thereby improving model interpretability and enabling cross-study comparison. For example, the evolutionary classification of >13 000 proteins across 79 000 *Salmonella enterica* strains demonstrated that nearly half of the domains associated with pathogenicity islands lacked prior sequence annotation. Many clinically enriched proteins were grouped into novel pathogenicity islands potentially associated with virulence and antibiotic biosynthesis, underscoring the latent structural diversity within pathogen genomes.

Overall, structural and deep genomic modeling represents a shift toward multi-scale reasoning in AMR prediction through the integration of nucleotide-, protein-, and phenotype-level information into unified analytical frameworks. By combining methods such as evolutionary classification systems, domain-adaptive attention mechanisms, and AlphaFold-derived embeddings, researchers have developed structure-informed pipelines that capture both the biophysical and evolutionary determinants of resistance. These advances have paved the way for transformer-based and foundation-level models that incorporate structural awareness into contextualized sequence reasoning.

**Key transition: from structural representation to contextual reasoning**

Structural and deep genomic modeling plays a central role in AMR prediction by linking protein conformation to functional expression. Building on this foundation, transformer and foundation architectures extend these advances by directly learning contextual relationships from sequence and structure, thereby enabling a unified and interpretable framework for multi-omics understanding.

#### Transformer-based and applied LLM frameworks for AMR

The emergence of transformer architectures has redefined the landscape of AMR modeling by introducing scalable context learning and self-attention mechanisms capable of capturing long-range dependencies across biological sequences. Models such as DNABERT, ProkBERT, and Qwen2 have demonstrated the ability to encode complex genomic relationships, learning hierarchical biological syntax analogous to linguistic grammar [73–75]. By tokenizing nucleotide or amino acid sequences, these models convert biological data into structured representations suitable for downstream prediction and classification tasks. Fine-tuned variants have been successfully applied to AMR-related challenges such as genome-wide resistance-gene identification, regulatory-element extraction, and promoter or phage detection. Their robust cross-species generalization has also enabled effective zero- and few-shot learning in microbial genomics.

Beyond sequence-level modeling, LLMs have increasingly been applied to broader AMR intelligence tasks that integrate molecular, textual, and epidemiological information. For example, transformer-based architectures have shown promise in real-time pathogen surveillance, food-hazard detection, and outbreak tracing through hierarchical machine learning frameworks and RoBERTa-based systems [76, 77]. These implementations exemplify applied LLM pipelines that bridge biological knowledge with public health analytics, providing interpretable and adaptive responses to emerging infectious threats.

The convergence of structural, genomic, and contextual learning has led to the development of data-efficient and interpretable foundation models capable of reasoning across multiple modalities. Transformer-based systems are evolving from static prediction toward causal inference and evidence-driven decision support as they increasingly incorporate domain adaptation, multimodal fusion, and uncertainty quantification. This growing synergy between bioinformatics and language modeling reflects a methodological transition from task-specific algorithms to dynamic, generalizable frameworks capable of simultaneously processing biological, clinical, and environmental signals. This progression marks a critical turning point in computational AMR research. The realization of trustworthy, explainable, and clinically viable AMR intelligence will depend on integrating transformer and foundation models with biological priors, interpretability methods, and active-learning mechanisms.

**Insight: toward unified reasoning in AMR prediction**

Transformer-based and LLMs extend AMR prediction from isolated sequence analysis to holistic reasoning across genomic, structural, and epidemiological domains. Their strength lies in contextual understanding, enabling them to learn not only *what* mutations occur, but also *why* and *how* they lead to resistance. Future progress will depend on integrating biological priors and interpretability mechanisms to establish reliable foundation-level AMR intelligence.

## Comparative visualization and synthesis of AMR modeling paradigms

This section provides a comparative, visualization-driven synthesis of computational paradigms for AMR modeling. Rather than cataloging individual methods, the focus is on identifying structural shifts in modeling philosophy, from feature-engineered prediction toward multimodal, generative, causal, and agentic intelligence. By combining conceptual taxonomies, temporal timelines, and integrative schematics, this section highlights how advances in data availability, model architecture, and reasoning capability have collectively reshaped the computational landscape of AMR research.

The analysis proceeds along three complementary dimensions. First, it traces the temporal evolution of AMR modeling approaches, illustrating how classical machine learning gave way to deep learning, transformer-based models, and autonomous systems. Second, it synthesizes emerging integrative frameworks that unify omics data, language models, and structured knowledge into cohesive biological intelligence pipelines. Third, it examines how causal reasoning, evolutionary forecasting, and agentic decision-making extend AMR modeling beyond static prediction toward adaptive, intervention-aware systems.

Together, the visual and conceptual frameworks presented in this section provide a unifying lens through which to understand how AMR modeling is transitioning from isolated predictive tasks to foundation-level, reasoning-centered computational biology.

### Temporal evolution of computational paradigms for AMR prediction

**Figure 4:**
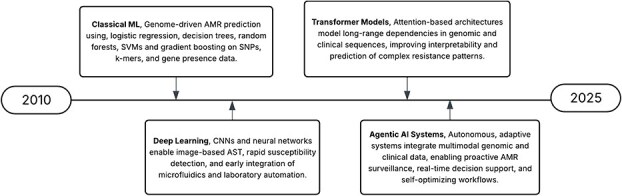
Temporal evolution of computational paradigms for AMR prediction. The timeline illustrates the progression from classical machine-learning approaches based on handcrafted genomic features, through deep learning and transformer-based models, to emerging agentic AI systems that integrate multimodal genomic and clinical data for adaptive surveillance and decision support.

Computational paradigms for AMR prediction have evolved substantially over the past decade, reflecting a clear shift toward increasingly sophisticated and autonomous systems [80]. This progression has been driven by the rapid expansion of available genomic and clinical data, advances in computational power, and the urgent need for fast and accurate AMR detection and management in clinical and public health settings [81, 82]. Early approaches primarily relied on classical machine-learning techniques operating on handcrafted genomic features, such as single nucleotide polymorphisms, *k*-mers, and gene presence–absence profiles, whereas later developments introduced deep learning architectures capable of capturing complex sequence-level and image-based susceptibility patterns [52, 83–85]. As illustrated in Fig. 4, this temporal evolution reflects a transition from feature-engineered, genome-driven models to deep-learning frameworks and, more recently, to transformer-based and agentic AI systems that integrate multimodal genomic, clinical, and epidemiological data for adaptive surveillance and real-time decision support.

#### Early application of machine learning

In the early stages of AMR research, machine-learning methods were increasingly applied to identify resistance phenotypes and their underlying genetic mechanisms. By 2016, researchers at PATRIC had developed AdaBoost-based classifiers to predict carbapenem resistance in *Acinetobacter baumannii*, methicillin resistance in *Staphylococcus aureus*, and beta-lactam and co-trimoxazole resistance in *Streptococcus pneumoniae*, achieving prediction accuracies ranging from 88% to 99%. These classifiers established an early framework for species-specific AMR phenotype prediction and genomic feature analysis within annotation platforms such as RAST and PATRIC. During this period, the increasing availability of large-scale datasets, driven by advances in next-generation sequencing technologies and the integration of electronic health records, enabled the broader adoption of machine-learning approaches in AMR studies.

#### Advancements in machine learning and genomic data integration

Between 2017 and 2020, the application of machine-learning techniques to AMR prediction expanded considerably, with increasing emphasis on integrating genomic data to enable more precise and scalable predictions. During this period, studies began employing decision tree-based models to predict AMR phenotypes directly from sequence data, including features derived from core genes not traditionally associated with resistance mechanisms. For example, as early as 2017, machine learning combined with structural analysis was used to identify evolutionary signatures of AMR across 13 antibiotics in *Mycobacterium tuberculosis*. By 2018 and 2019, machine-learning models had been further developed to predict observed AMR in agricultural non-typhoidal *Salmonella enterica* serovars. In 2020, a novel bioinformatics framework known as VAMPr (Variant Mapping and Prediction of Antibiotic Resistance) was introduced. VAMPr curated >3000 bacterial genomes spanning nine species, together with associated AMR phenotypes across 29 antibiotics, and constructed 93 association and prediction models demonstrating high predictive performance.

### Integrative frameworks for multimodal biological intelligence

**Figure 5:**
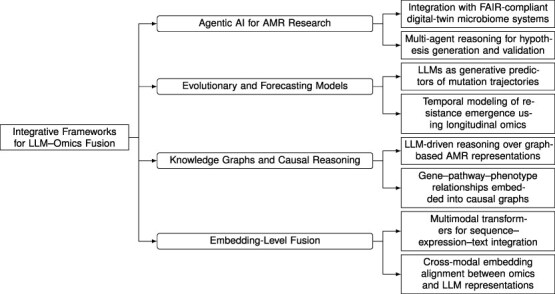
Taxonomy of integrative frameworks uniting LLMs and omics analytics. The hierarchy delineates four methodological paradigms shaping the emerging field of LLM–omics fusion: (i) **Embedding-Level Fusion**, which aligns omics and linguistic embeddings through multimodal transformer architectures; (ii) **KGs and Causal Reasoning**, which embed structured biological relationships into LLM-based inference; (iii) **Evolutionary and Forecasting Models**, which apply LLMs to simulate mutation trajectories and resistance emergence; and (iv) **Agentic AI for AMR Research**, which employs multi-agent reasoning frameworks for autonomous discovery, simulation, and validation. Together, these paradigms illustrate how foundational LLM technologies can evolve into biologically grounded intelligence systems that connect molecular data with contextual reasoning.

Computational biology is undergoing a major transformation driven by the convergence of multi-omics analytics and LLMs. Recent advances indicate that transformer-based architectures can jointly model relationships among genomic, proteomic, transcriptomic, and phenotypic signals while also enabling text-grounded reasoning over biomedical knowledge. Figure 5 summarizes this convergence as a taxonomy of four integrative directions: embedding-level fusion, KGs and causal reasoning, evolutionary and forecasting models, and agentic AI for AMR research. To enhance structural clarity and maintain a continuous narrative, we consolidate the discussion into four coherent thematic blocks aligned with this taxonomy, spanning representation-level fusion, mechanistic knowledge integration, temporal forecasting, and feedback-enabled autonomous reasoning.

#### Embedding-level fusion

Embedding-level fusion provides the representational foundation for multimodal biological intelligence by mapping heterogeneous omics signals and biomedical language into a shared latent space. In a typical pipeline, modality-specific encoders transform genomic sequences, protein structures, molecular graphs, expression profiles, and biomedical text into embeddings that can be jointly modeled. These embeddings are subsequently integrated through hierarchical fusion strategies that progress from alignment- and projection-based correspondence to attention-mediated cross-modal interaction and, ultimately, generative transformer fusion. Alignment-based methods establish correspondence between modalities, attention-based mechanisms learn conditional dependencies between molecular and textual features, and generative LLM fusion supports synthesis by producing text-grounded hypotheses or molecular candidates from integrated representations. This unified representation enables downstream tasks including AMR prediction, drug discovery, protein function annotation, and disease forecasting, while improving cross-domain generalization through the learning of modality-invariant features across heterogeneous inputs.

**Figure 6:**
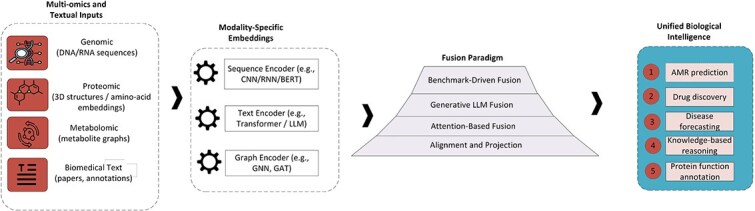
Information flow in LLM–omics embedding fusion. Multi-omics and textual inputs, including genomic, proteomic, metabolomic, and biomedical text data, are first processed by modality-specific encoders such as CNNs, RNNs, GNNs, and transformers. These embeddings are subsequently integrated through a hierarchical fusion paradigm that progresses from alignment- and projection-based correspondence (Cluster A) to attention-based cross-modal integration (Cluster B), generative LLM fusion frameworks (Cluster C), and benchmark-driven multimodal systems (Cluster D). The resulting unified latent representations enable biologically interpretable reasoning across diverse downstream applications, including AMR prediction, protein function annotation, drug discovery, disease forecasting, and knowledge-based inference.

This progression is summarized in Fig. 6, which organizes the fusion pipeline from modality-specific encoding through hierarchical integration to downstream biological inference.

Representative frameworks illustrate this evolution from correspondence learning to reasoning and synthesis. Sequence–text alignment models map proteins to functional descriptions, enabling interpretable annotation and function discovery, while graph-based and attention-based fusion extends cross-modal modeling to relational settings such as drug–target, gene–disease, and microbe–drug association discovery. Generative multimodal transformers further shift fusion from association to synthesis by jointly modeling molecular graphs, omics sequences, and biomedical text within shared backbones. ProtCLIP [86] and Prot2Text [87] exemplify sequence–text alignment; iADRGSE [88] and GSAMDA [89] demonstrate graph-based fusion; CFAGO [90], MOSGAT [91], and DMMAFS [92] illustrate attention-based integration; BioMedGPT [93], BioT5 [94], and GexMolGen [95] represent generative multimodal backbones; and applied systems such as C0mic [96], DeePROG [97], and AntiViralDL [98] translate these ideas into clinical and epidemiological settings. Across this spectrum, the central mechanism remains consistent: fusion transforms heterogeneous biological evidence into unified representations that are both predictive and interpretable.

#### Knowledge graphs and causal reasoning

Whereas fusion produces shared representations, KGs provide explicit biomedical structure by encoding gene–pathway–phenotype relationships, molecular interactions, and clinical entities. When coupled with LLMs, KGs evolve from static repositories into dynamic reasoning substrates that support multi-hop inference, evidence tracing, and causal hypothesis generation. Ontology-guided frameworks established the foundation for ontology-consistent inference, followed by AMR-focused KGs that integrate resistance mechanisms and clinical context to enable actionable reasoning over antibiotic–gene associations. BioKG [99] and DeepGOZero [100] provide ontology-guided foundations, whereas AMR-KG [101] and DR.KNOWS [102] extend this paradigm toward resistance modeling.

**Figure 7:**
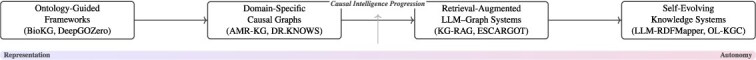
**Evolution of KG reasoning in biomedical intelligence.** This conceptual trajectory illustrates the methodological progression from ontology-guided modeling to causal graph reasoning, retrieval-augmented systems, and self-evolving LLM-integrated knowledge architectures. The gradient bar represents the transition from static representation to autonomous reasoning, while the upward arrow signifies the increasing causal and interpretive capacity of modern AMR intelligence frameworks.

More recent retrieval-augmented KG–LLM systems treat graphs as structured memory modules, improving factual grounding and interpretability through explicit traversal, subgraph retrieval, and provenance-aware reasoning. KG-RAG [103], KRAGEN [104], and ESCARGOT [105] exemplify this direction. Finally, ontology-aligned semantic mediation approaches improve interoperability across fragmented biomedical repositories by connecting RDF ontologies, embedding-driven mapping, and contextual retrieval. DrKGC [106], OL-KGC [107], and LLM-RDFMapper [108] illustrate this progression. Figure 7 summarizes this methodological trajectory from ontology-guided frameworks to retrieval-augmented and self-evolving knowledge systems. Overall, KG–LLM integration operationalizes interpretability by linking predictions to explicit biomedical evidence, thereby enabling reasoning that is both traceable and biologically grounded.

#### Evolutionary and forecasting models

**Figure 8:**
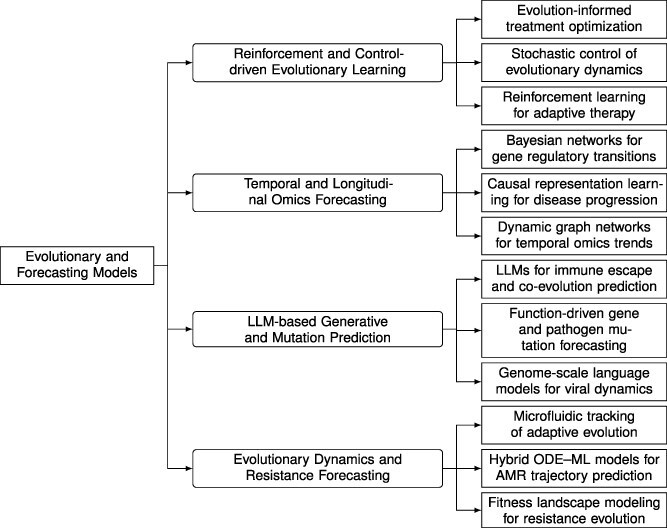
Taxonomy of evolutionary and forecasting models in biomedical systems. The hierarchy groups studies into four conceptual clusters: (i) **Evolutionary Dynamics and Resistance Forecasting**, covering fitness landscapes and adaptive resistance trajectories; (ii) **LLM-based Generative and Mutation Prediction**, focusing on mutation forecasting and co-evolutionary modeling; (iii) **Temporal and Longitudinal Omics Forecasting**, addressing dynamic molecular and clinical processes using causal and graph-based frameworks; and (iv) **Reinforcement and Control-driven Evolutionary Learning**, applying control-theoretic and reinforcement principles to optimize adaptive therapy and resistance management.

Evolutionary and forecasting frameworks extend multimodal intelligence from cross-sectional inference to temporally grounded prediction by focusing on how biological systems adapt under selective pressure. As summarized in Fig. 8, this direction spans four complementary clusters: evolutionary dynamics and resistance forecasting, LLM-based generative mutation prediction, temporal and longitudinal omics forecasting, and reinforcement- and control-driven evolutionary learning. Across these clusters, the shared objective is to connect mechanistic understanding with data-driven forecasting in order to anticipate resistance emergence, pathogen evolution, and treatment outcomes.

Representative studies operationalize this direction at multiple scales, from molecular co-evolution and metagenomic annotation to population-level variant surveillance and adaptive intervention design. DeepARG [48] and FGeneBERT [109] support resistance-gene discovery and metagenomic annotation; GenSLM [110] and GenoSig [111] enable genome-scale surveillance and lineage tracking; and reinforcement/control frameworks such as the Antibiotic Cycling Platform [112] connect evolutionary dynamics to intervention policies. Temporal deep learning and causal forecasting further extend these capabilities by modeling time-resolved omics transitions, microbial dynamics, and disease trajectories using dynamic graphs, temporal transformers, and Bayesian networks, including settings characterized by missing data and irregular sampling. Collectively, evolutionary forecasting provides the temporal competence needed to move from describing resistance to anticipating and shaping it.

#### Agentic AI for AMR research

**Figure 9:**
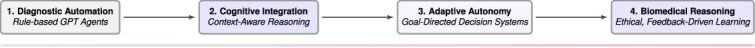
**Conceptual trajectory of agentic AI in AMR research.** The framework visualizes the progressive shift from diagnostic automation to biomedical reasoning, highlighting the integration of autonomy, feedback, and ethical cognition within multimodal medical AI systems.

Agentic AI frameworks introduce autonomy and feedback into multimodal bioinformatics pipelines, enabling systems that plan, retrieve evidence, interact with tools, and iteratively refine hypotheses under explicit constraints. In AMR, this supports end-to-end workflows that connect phenotype interpretation, guideline-constrained reasoning, literature grounding, and simulation-based validation within FAIR-compliant infrastructures. Empirical studies indicate that guideline-grounded LLM agents can support well-defined diagnostic subtasks, including phenotype pre-classification under standardized microbiology guidelines. Surveys and industrial deployments likewise highlight agentic architectures that integrate curated databases, literature retrieval, and predictive models within auditable, end-to-end biomedical workflows. Giske *et al.* [118] provide a quantitative evaluation of GPT-4-based assistance for $\beta$-lactamase phenotype pre-classification under EUCAST guidelines; Acharya *et al.* [119] survey agentic AI principles; and APPA [120] illustrates domain-grounded agentic automation in pharmaceutical research. As conceptualized in Fig. 9, agentic systems follow a trajectory from diagnostic automation to context-aware, goal-directed autonomy, with increasing requirements for transparency, traceability, and governance.

**Key insight: a unified view of multimodal biological intelligence**

The four directions in Fig. 5 form a coherent progression: fusion provides shared representations, knowledge graphs provide mechanistic structure, forecasting provides temporal competence, and agentic systems provide autonomy and feedback. This progression offers a practical blueprint for foundation-level bioinformatics systems that are not only accurate but also interpretable, evolvable, and aligned with reproducible scientific workflows.

## Comparative analysis of the literature

This section provides a structured comparative synthesis of the computational literature on AMR, with an emphasis on data modalities, preprocessing strategies, integration pipelines, and emerging evolutionary modeling frameworks. Rather than evaluating individual studies in isolation, the analysis focuses on identifying cross-study trends, methodological commonalities, and persistent gaps that shape the current AMR modeling landscape.

Across the reviewed literature, a clear hierarchy emerges in both data availability and analytical maturity. Genomics remains the dominant modality, supported by large-scale public repositories and standardized pipelines, whereas transcriptomic, proteomic, metabolomic, and host–pathogen interaction datasets appear less frequently and are often constrained by smaller sample sizes and limited harmonization. The comparative tables presented in this section summarize representative datasets, preprocessing practices, and integration strategies, thereby enabling systematic evaluation of how data diversity and methodological design influence predictive modeling outcomes.

Collectively, this comparative analysis highlights a transition from static, single-layer modeling toward integrative, multi-omics, and temporally informed frameworks. However, it also reveals substantial variability in preprocessing rigor, fusion strategies, and reproducibility reporting, underscoring the need for standardized, FAIR-compliant pipelines and evaluation benchmarks to support scalable and interpretable AMR intelligence.

### Multi-omics foundations for AMR prediction

**Figure 10:**
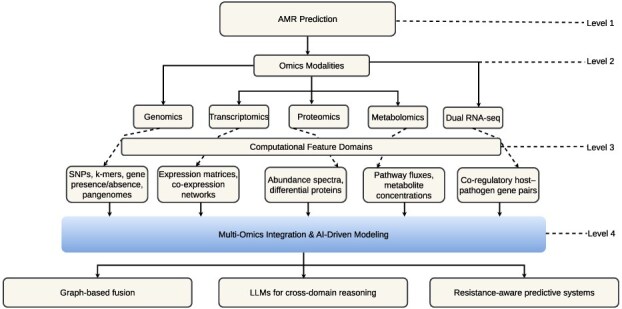
*Hierarchical taxonomy of multi-omics data integration for AMR prediction.* The taxonomy illustrates the multilevel relationships among genomic, transcriptomic, proteomic, metabolomic, and dual RNA-seq modalities (Level 2), their associated computational feature domains (Level 3), and downstream AI-driven modeling paradigms (Level 4). It emphasizes how diverse omics layers converge into unified feature representations that enable integrative reasoning and predictive modeling in AMR research.

The hierarchical taxonomy illustrated in Fig. 10 outlines a multilevel framework for AMR prediction. It traces the analytical progression from broad biological modalities (Level 2) to computational feature domains (Level 3) and downstream AI-driven modeling paradigms (Level 4). This structure conceptualizes how genomics, transcriptomics, proteomics, metabolomics, and dual RNA-seq collectively inform integrative AMR modeling, uniting static and dynamic molecular signals within reasoning-based, resistance-aware predictive systems. The taxonomy underscores that AMR prediction increasingly depends on cross-modal synthesis, in which distinct omics datasets converge into transparent, AI-augmented inference pipelines.

WGS remains the cornerstone of AMR prediction, linking genetic determinants such as SNPs, plasmid replicons, and accessory genes to phenotypic resistance. Large-scale repositories (e.g. NARMS, NCBI, and CIPARS) have supported population-level analyses (Hu *et al.* [121]; Xu *et al.* [122]), thereby forming the foundation for genotype–phenotype association studies. Representative genomic datasets and key methodological trends are summarized in Table 5, highlighting how population-scale sequencing and regional surveillance jointly shape current predictive frameworks.

**Table 4:** Representative frameworks for evolutionary and forecasting modeling in resistance and pathogen evolution studies. Each framework integrates temporal, generative, or reinforcement-based reasoning to predict evolutionary trajectories and adaptive responses across biological systems.

Framework/Study	Model type	Data modality	Forecasting	Application
*Infectious Disease Research* (2024) [113]	Review of protein, genomic, and multimodal LLMs	Genomic, proteomic, and pathogen sequence data	Evolutionary surveillance and host–pathogen modeling using large-scale embeddings	Pathogen evolution, surveillance, and therapeutic discovery
*DeepARG* (2023) [48]	DL model using dissimilarity matrices for ARG prediction	Metagenomic and genomic sequence data	Neural network-based prediction of antibiotic resistance gene categories	ARG detection, resistance monitoring, and evolutionary surveillance
*Pathogen Evolution Forecasting* (2025) [114]	Review of AI- and language-model-based viral evolution prediction	Genomic, epidemiologic, and immunologic data	Deep learning- and LM-based forecasting of viral mutation trajectories	Viral evolution, immune evasion, and pandemic preparedness
*FGeneBERT* (2025) [109]	Pretrained gene language model for metagenomic data	Protein-based gene representations and metagenomic sequences	Masked gene modeling with triplet contrastive learning for sequence–function understanding	Functional annotation, microbial evolution, and environmental metagenomics
*L2 $\beta$-Lactamases* (2025) [115]	Structural and sequence data of class A $\beta$-lactamases (L2, SME-1, KPC-2)	Molecular dynamics trajectories and protein structure features	CVAE and BindSiteS-CNN for dynamic clustering and co-evolutionary inference	Enzyme evolution, active-site dynamics, and AMR mechanism discovery
*Antibiotic Cycling Platform* (2025) [112]	Empirical fitness landscapes of *E. coli* under $\beta$-lactam antibiotics	Evolutionary simulation data and population fitness metrics	Reinforcement learning for adaptive feedback control and policy optimization	Drug cycling, resistance suppression, and evolutionary control of pathogens
*Metabolic Fitness Landscape Model* (2024) [116]	Experimental growth data and genomic profiles of *E. coli* resistance mutants	Metabolic flux, fitness, and drug-response parameters	Quantitative Pareto-surface modeling of resistance–growth trade-offs	Prediction of evolutionary trajectories and metabolic adaptation under drug stress
*GenSLM* (2025) [110]	110M prokaryotic gene sequences and 1.5M SARS-CoV-2 genomes	Genomic embeddings and evolutionary mutation data	LLM-based genome-scale modeling for variant detection and evolutionary forecasting	SARS-CoV-2 variant identification and pandemic evolution tracking
*GenoSig* (2024) [111]	SARS-CoV-2 genome sequences across multiple global clades	Di- and tri-nucleotide frequency features with ML/DL embeddings	Alignment-free deep learning and random forest models for taxonomic and clade prediction	Genomic lineage classification and global variant-dynamics monitoring
*Evolution-Informed Cancer Therapy* (2023) [117]	Multi-omics and clinical data from heterogeneous cancer cohorts	Gene signatures, computational drug-response models, and ecological dynamics	Evolutionary and adaptive-therapy modeling with predictive biomarkers	Controlling therapeutic resistance through eco-evolutionary and adaptive treatment strategies

**Table 5:** Representative genomics-based resources and features used in AMR prediction. This table highlights the breadth of genomic approaches applied to *Salmonella* and other bacterial pathogens, ranging from population-scale sequencing efforts (e.g. Hu *et al*. [121]; Xu *et al*. [122]) that enable robust genotype–phenotype mapping, to regional surveillance studies (e.g. Bayliss *et al*. [77]; Bharat *et al*. [135]) capturing epidemiological and geographical variation. Comparative analyses extend beyond *Salmonella* (e.g. Gao *et al*. [136]; Nguyen *et al*. [137]), illustrating the transferability of genomic features such as k-mer profiles and pangenome variation. Smaller-scale studies (e.g. Pornsukarom *et al*. [138]; Tay *et al*. [139]; Carroll *et al*. [140]) underscore the utility of WGS for clinical diagnostics and outbreak investigations. Together, these efforts show that genomics remains the dominant modality for AMR prediction, though its static nature highlights the need for integration with transcriptomic, proteomic, and metabolomic layers.

Study	Dataset	Feature type	Sample sze	Publicly available
You *et al*. [75]	NARMS, NCBI	Accessory genes and SNPs	1167 isolates	✓
Bayliss *et al*. [77]	UKHSA Enteritidis	Unitig presence/absence patterns; hierarchical region/subregion/country classifier	2313 isolates	✓
Wu *et al*. [141]	Cornell Food Safety Lab	AMR and virulence gene profiles, serotype prediction	69 isolates	✓
Woh *et al*. [142]	NCBI	Resistome + phenotype whole-genome features	788 isolates	✓
Bharat *et al*. [135]	CIPARS	AMR genes + point mutations (Staramr)	1321 isolates	✓
Ayoola *et al*. [69]	NARMS	K-mer counts (10–20-mers) + gene features for MIC prediction	4500 isolates	✓
Pornsukarom *et al*. [138]	NCBI BioProject	AMR genes, virulence genes, plasmid replicons; core genome SNP + feature frequency profiling	200 isolates	✓
Tay *et al*. [139]	Sri Lanka surveillance	Serotyping; sequence type + plasmid replicon + AMR gene detection	73 isolates	✓
Carroll *et al*. [140]	NY/WA cattle and clinical isolates	AMR genes versus phenotypic resistance (WGS-based)	90 isolates	✓
Benefo *et al*. [143]	Chicken-meat *Salmonella*	WGS genomes + AST phenotype annotations	405 isolates	✓
Xu *et al*. [122]	PATRIC MTB + external cohorts	SNP genotypes + AST phenotype annotations	5739 isolates	✓
Hu *et al*. [121]	PATRIC	Genomic features; AST phenotype annotations; quality metrics (sequence type, contig number, genome length, PATRIC quality scores)	31 195 isolates	✓
Gao *et al*. [136]	Clinical *A. baumannii*	K-mer features (11-mers) + MIC phenotype annotations	459 isolates	✓
Liu *et al*. [144]	Clinical *S. aureus*	Gene presence/absence; SNPs; k-mer features + AST phenotype annotations	3979 isolates	✓
Nguyen *et al*. [137]	Public *E. coli, K. pneumoniae*	Pangenome: gene presence/absence; amino-acid variants; k-mer profiles of AMR-gene clusters + AST phenotype	6487 *E. coli*, 2774 *K. pneumoniae*	✓

Complementing genomics, transcriptomic analyses capture the temporal reprogramming of bacterial gene expression under antimicrobial exposure. Transcriptomic resources constitute the primary dynamic layer complementing genomic variation in AMR modeling by capturing condition-specific regulatory responses to antimicrobial stress (Table 6). As summarized in Table 6, studies by Khaledi *et al.* [123], Li *et al.* [124], and Qin *et al.* [125] have revealed distinct transcriptional signatures associated with resistance, although limited standardization and dataset fragmentation still constrain cross-condition generalization.

**Table 6:** Transcriptomics-based studies and resources in AMR research. The table highlights the diversity of transcriptomic investigations, ranging from clinical isolate profiling in *P. aeruginosa* (e.g. Khaledi *et al*. [123]; Shahreen *et al*. [145]) to experimental studies in *A. baumannii* and *N. gonorrhoeae* (e.g. Qin *et al*. [125]; Manoharan-Basil *et al*. [146]). Meta-transcriptomic and ecological approaches extend beyond pathogens (Marcelino *et al*. [147]), while industrial stress conditions (Yang *et al*. [148]) broaden the scope of regulatory contexts analyzed. Although these studies reveal valuable gene expression signatures and regulatory programs, transcriptomic datasets remain fragmented and underrepresented compared with genomics, underscoring the need for larger, benchmark-ready repositories.

Study	Dataset	Condition	Sample type	Data availability
Khaledi *et al*. [123]	Clinical *Pseudomonas aeruginosa* isolates ($n=135$)	Resistance versus susceptible to fluoroquinolones, aminoglycosides, $\beta$-lactams	Clinical isolates	✓
Khaledi *et al*. [149]	Clinical *P. aeruginosa* isolates	Combined genomic and transcriptomic profiling for AMR prediction	Clinical isolates	✓
Li *et al*. [124]	*Serratia marcescens* strains	Comparative transcriptomics under antibiotic exposure	Laboratory isolates	✓
Qin *et al*. [125]	*Acinetobacter baumannii* MDR strains	Transcriptomic response under different antibiotic treatments	Clinical isolates	✓
Marcelino *et al*. [147]	Wild bird gut microbiomes (Australia and Antarctica)	Meta-transcriptomic analysis of expressed antimicrobial resistance genes (ARGs)	Environmental microbiome samples	✓
Manoharan-Basil *et al*. [146]	Laboratory reference strain *N. gonorrhoeae* WHO P	Ceftriaxone tolerance induced by intermittent high-dose exposure (10$\times$ the MIC, over multiple days)	Laboratory isolates ($n=12$ tolerant + $n=3$ controls)	✓
Harrington *et al*. [150]	Pig lung infection model, *P. aeruginosa* biofilms	Transcriptome analysis during *in vivo* biofilm growth	*In vivo* biofilm samples	✓
Jeukens *et al*. [151]	Clinical *P. aeruginosa* isolates	Transcriptomic contribution to AMR prediction with WGS	Clinical isolates	✓
Shahreen *et al*. [145]	Clinical *P. aeruginosa* isolates (n$=414$)	Minimal gene signatures predictive of AMR	Clinical isolates	✓
Yang *et al*. [148]	Industrial vinegar fermentation (*Komagataeibacter europaeus*)	Transcriptomic profiling under high acetic acid stress (8%–12%)	Industrial submerged fermentation samples (initial, mid, final; two replicates each)	✓

Beyond transcriptional regulation, functional adaptations at the protein and metabolic levels are summarized across representative studies in Table 7. Proteomic and metabolomic layers provide a complementary view of downstream regulatory and metabolic adaptation. Table 7 compiles representative resources showing how proteomic analyses by Liu *et al.* [126] and Safi *et al.* [127] quantify protein abundance under stress, whereas metabolomic studies by Overton *et al.* [128] and Li *et al.* [129] trace perturbations in pathway fluxes and metabolite concentrations linked to persistence and tolerance phenotypes.

**Table 7:** Proteomics and metabolomics resources applied in AMR research. Studies span intracellular proteomic surveys (e.g. Liu *et al*. [126]), stress-response profiling (Kim *et al*. [152]; Huang *et al*. [153]), and comparative analyses of resistant versus nonresistant strains (Safi *et al*. [127]; Yasin *et al*. [154]). Metabolomics complements these efforts, capturing environmental and stress-induced metabolic changes (Overton *et al*. [128]; Li *et al*. [129]). Systems-level studies such as Kim *et al*. [155] integrate metabolomics into multi-omics frameworks of host–pathogen interactions. While these approaches uncover critical functional adaptations, the field remains constrained by small datasets and limited standardization, underscoring the need for expanded proteomic and metabolomic resources for AMR prediction.

Study	Omics type	Focus	Sample size	Data availability
Overton *et al*. [128]	Metabolomics	Metabolomic profiles of MDR *S. Typhimurium*	$\times$	✓
Liu *et al*. [126]	Intracellular *Salmonella enterica* (infected epithelial cells)	Comprehensive proteomic survey; 3300 proteins identified, 100 significantly altered during intracellular replication	$\times$	✓
Huang *et al*. [153]	Proteomics	Regulation of immune pathways in *Salmonella* under stress	$\times$	✓
Safi *et al*. [127]	Proteomics	Differential proteomics of drug-resistant versus nonresistant *S. Typhi*	43 isolates	✓
Yasin *et al*. [154]	Proteomics	Up-regulated metabolic proteins in MDR and XDR *S. Typhi* blood isolates	6 clinical isolates (3 MDR + 3 XDR)	Upon requests
Li *et al*. [129]	Metabolomics	Metabolite profiling of *S. Enteritidis* under desiccation (24 h) and skimmed milk powder storage (3 months)	9 samples (3 biological replicates $\times$ 3 conditions: baseline, 24 h desiccation, 3-month SMP storage)	✓
Kim *et al*. [155]	Multi-omics	Host–pathogen interaction during *Salmonella* infection (mouse model)	44 fecal samples (11 mice $\times$ 4 timepoints)	✓
Kim *et al*. [152]	Proteomics	Quantitative proteomic profiling of *S. Enteritidis* under oxidative stress (H$_{2}$O$_{2}$ exposure); 76 proteins modulated; validation in macrophages and spleen	$\times$	✓

Extending further, dual RNA-seq provides a bidirectional view of host–pathogen interactions by capturing simultaneous transcriptional responses from both organisms. Representative datasets are summarized in Table 8. Studies by Westermann *et al.* [130] and Kocabaş *et al.* [131] integrate host immune responses and bacterial adaptation within co-regulated gene networks. Together, these resources form a comprehensive empirical foundation for reasoning-centered, AI-driven AMR prediction. As conceptualized in Fig. 10, multi-omics integration bridges static genomic variation with dynamic molecular adaptation, thereby advancing interpretable, data-centric, and FAIR-compliant antimicrobial research.

**Table 8:** Representative dual RNA-seq and host–pathogen interaction datasets applied in *Salmonella* AMR and infection biology research. These studies integrate bacterial and host transcriptomic profiles to capture dynamic co-regulation during infection. Early dual RNA-seq efforts (e.g. Westermann *et al*. [130]; Avraham *et al*. [156]) characterized simultaneous gene expression shifts in macrophages infected with *S. Typhimurium*, while recent single-cell approaches (e.g. Avital *et al*. [157]; Saliba *et al*. [158]) have resolved heterogeneity at cellular resolution. Integrated frameworks (e.g. Kocabaş *et al*. [131]) and infection-lifestyle profiling studies (e.g. García-del Portillo *et al*. [159]; Westermann and Vogel [160]) extend these analyses to metabolic network modeling and cross-species interactions.

Study	Approach	Host system	Bacterial strain	Dual profiling
Westermann *et al*. [130]	Dual RNA-seq	Murine macrophages (bone-marrow derived)	*S. Typhimurium* SL1344	✓
Avital *et al*. [157]	scDual-seq (single-cell)	Mouse macrophages	*S. Typhimurium*	✓
Saliba *et al*. [158]	Single-cell RNA-seq	Murine macrophages	*S. Typhimurium*	✓
Avraham *et al*. [156]	Single-cell dual RNA-seq	Murine bone marrow macrophages	*Salmonella* Typhimurium (ATCC 14028s, SL1344, PhoP mutants)	✓
Aulicino *et al*. [161]	Dual RNA-seq	Human dendritic cells	*S. Typhi*	✓
Kocabaş *et al*. [131]	Dual RNA-seq + integrated pathogen-host metabolic network modeling	HeLa human epithelial cells infected with *S. Typhimurium* SL1344	*S. Typhimurium* SL1344	✓
García-del Portillo *et al*. [159]	RNA-seq (infection lifestyle profiling)	Human epithelial cell lines	*S. enterica*	✓
Westermann & Vogel [160]	Dual RNA-seq (review + case studies)	Murine and human systems	*S. Typhimurium*	✓
Westermann *et al*. [160]	Review of dual RNA-seq + summary of published host–pathogen datasets	Human cell culture (including HeLa epithelial cells, porcine macrophages)	Multiple S. *Typhimurium* strains surveyed	✓

Beyond improving predictive accuracy, multi-omics studies often provide biologically interpretable insights by linking resistance phenotypes to coherent functional programs. For example, integrative latent-variable frameworks can reveal pathway-level convergence across the transcriptome, proteome, and metabolome, highlighting metabolic reprogramming and immune-related stress signals that are not recoverable from static gene presence alone [132]. Systems-scale multi-omic analyses further suggest that resistance and persistence may be shaped by host–pathogen–microbiome interactions, in which inflammation-driven ecological shifts and metabolite availability modulate bacterial survival strategies [133]. Time-resolved integration additionally supports mechanistic interpretation by exposing phase-specific metabolic plasticity and context-dependent pathway usage across infection or environmental transitions [134]. Together, these findings motivate framing multi-omics AMR modeling not only as a performance enhancement, but also as a mechanism-aware approach capable of generating testable hypotheses about regulatory adaptation, metabolic constraints, and tolerance-associated states under antimicrobial pressure.

### Preprocessing pipelines for multi-omics modeling

To assess methodological consistency and reproducibility across multi-omics AMR studies, preprocessing practices are summarized at both the data level (Table 9) and the integration or modeling level (Table 10). Preprocessing constitutes a foundational step in multi-omics modeling, ensuring that the heterogeneous characteristics of genomic, transcriptomic, proteomic, and metabolomic data are harmonized prior to integration. Systematic biases, noise, and missing information can propagate through downstream analyses and compromise model interpretability if they are not addressed appropriately. To evaluate methodological consistency across the literature, representative *Salmonella* multi-omics studies were examined and categorized according to two complementary levels of preprocessing: (i) data-level procedures applied before integration (Table 9), and (ii) integration- or modeling-level strategies encompassing feature selection, fusion, and optimization methods (Table 10).

**Table 9:** Data-level preprocessing steps adopted across representative multi-omics *Salmonella* modeling studies. Each column indicates whether a given preprocessing step was applied before data integration.

Paper	Norm.	Log Transf.	Imput.	Outlier Rem.	Transform.	Scaling	Batch Corr.
Bolinger *et al*. [162]	✓	✓	✓	✓	✓	$\times$	$\times$
Mukherjee *et al*. [163]	✓	✓	✓	$\times$	✓	✓	✓
Jendoubi *et al*. [164]	✓	✓	✓	$\times$	✓	✓	✓
Nguyen *et al*. [165]	✓	$\times$	✓	$\times$	✓	✓	$\times$
Pasat *et al*. [166]	✓	$\times$	✓	✓	✓	✓	✓
Rohart *et al*. [167]	✓	✓	$\times$	$\times$	✓	✓	$\times$
Marwah *et al*. [168]	✓	✓	$\times$	✓	✓	✓	✓
Chierici *et al*. [169]	✓	✓	$\times$	$\times$	✓	✓	$\times$
Argelaguet *et al*. [170]	✓	✓	$\times$	$\times$	✓	✓	✓
Madrid–Márquez *et al*. [171]	✓	✓	✓	✓	✓	✓	✓
Planell *et al*. [172]	✓	✓	$\times$	✓	✓	✓	✓
Misra *et al*. [173]	✓	✓	✓	✓	✓	✓	✓
Kohl *et al*. [174]	✓	✓	✓	✓	✓	✓	✓

*Note:* ✓, applied; $\times$, not applied. Column abbreviations: **Norm.**, normalization; **Log Transf.**, logarithmic transformation; **Imput.**, imputation of missing values; **Outlier Rem.**, outlier removal; **Transform.**, other data transformations (e.g. variance-stabilizing, Box–Cox, dimensionality reduction); **Scaling**, feature-wise standardization or auto/Pareto scaling; **Batch Corr.**, batch effect correction (e.g. ComBat, sva, EigenMS). Normalization refers to distribution-level adjustments such as TPM/RPKM, quantile, or median scaling; scaling indicates per-feature variance normalization before modeling. Transformation denotes mathematical or latent-space modifications beyond simple log conversion (e.g. PCA, PLS, MOFA, Box–Cox). Batch correction mitigates site, platform, or experimental biases, while imputation covers statistical recovery of missing entries (kNN, BPCA, Random Forest, etc.). Abbreviated column names are used to maintain table compactness.

**Table 10:** Integration-level preprocessing and fusion strategies across multi-omics *Salmonella* studies. The table summarizes key computational methods used for omics integration.

Paper	Feature selection	Integration/Fusion method	Remarks
Yoon *et al*. [175]	✓	CLR Network (Sample-Matched)	**Network-based integration** linking transcriptomic and proteomic layers to infer *regulatory associations*.
Kang *et al*. [176]	✓	RNA-Seq + Genomic Integration (PanExplorer)	*Cross-omics DEG mapping* enabled genotype–phenotype association and detection of strain-level functional markers.
Cai *et al*. [177]	✓	Transcriptome–Proteome Correlation + Pathway Enrichment	**GO/KEGG-driven correlation** framework for multilayer alignment and *functional pathway convergence*.
Abdallah *et al*. [178]	✓	Multimodal ML Integration (Genomic + Phenotypic)	*Hybrid early–late fusion* using PCA, t-SNE, UMAP, logistic regression, and random forest; **AMR prediction** with interpretable ML ensemble.
Bai *et al*. [132]	✓	Multi-Omics Integration using DIABLO (Transcriptome + Proteome + Metabolome)	**Latent-variable modeling** revealed *glycolytic reprogramming* and ROS-mediated immune modulation distinguishing *S. Typhi* versus *S. Typhimurium*.
Deatherage Kaiser *et al*. [133]	✓	Multi-Omic Systems Analysis (Proteomics + Metabolomics + Glycomics + Metagenomics)	**Host–pathogen–commensal interplay**: inflammation-induced dysbiosis, metabolite shifts, and exploitation of *fucosylated glycans*.
Kokkinias *et al*. [134]	✓	Time-resolved Multi-Omics Integration (Metatranscriptomics + Targeted/Untargeted Metabolomics + 16S rRNA)	**Temporal fusion** revealed *phase-specific metabolic plasticity* and dietary effects on electron acceptor and carbon utilization pathways.
Chicco *et al*. [179]	✓	Methodological Framework for Multi-Omics Integration	**Best-practice principles** for standardization, metadata curation, redundancy control, and FAIR-compliant pipelines.

*Note:* ✓, applied; $\times$, not applied. Integration abbreviations: CLR, context likelihood of relatedness; DIABLO, Data Integration Analysis for Biomarker discovery using Latent variable approach for Omics; GO/KEGG, Gene Ontology and Kyoto Encyclopedia of Genes and Genomes; ML, machine learning; FAIR, Findable, Accessible, Interoperable, and Reusable data principles. Additional terms: DEG, differentially expressed gene; ROS, reactive oxygen species; “Early” and “Late” fusion refer to feature-level versus decision-level data integration; “Latent-variable modeling” denotes shared component inference across omic layers; “Metabolome” refers to the full set of metabolites quantified under experimental conditions.

Table 9 shows that normalization, transformation, and scaling are nearly universal across studies, reflecting their essential role in stabilizing feature distributions and ensuring cross-platform comparability. Logarithmic transformation and batch-effect correction are also frequently reported, particularly in frameworks using RNA-seq or proteomics data, whereas explicit outlier removal and imputation are less consistently described, suggesting substantial variation in the treatment of missingness and noise. These differences indicate that, although the field has converged on certain harmonization principles, important variability remains in the handling of data sparsity and batch heterogeneity.

At the integration level (Table 10), most studies employ structured feature selection followed by correlation-based or latent-variable fusion techniques such as DIABLO, CLR networks, and component-analysis models. More recent work emphasizes interpretable machine learning and multimodal embeddings to connect genotype, phenotype, and AMR traits. Collectively, these tables demonstrate a methodological shift from isolated preprocessing steps toward cohesive, model-aware normalization and integration frameworks. This synthesis underscores the growing emphasis on reproducibility, harmonization, and interpretability in contemporary *Salmonella* multi-omics research.

### Uncertainty quantification and robustness assessment

Quantifying and managing uncertainty are essential for developing reliable predictive models in microbial risk assessment and pathogen surveillance. Recent advances in computational modeling have increasingly incorporated probabilistic frameworks and uncertainty-aware inference to improve the robustness and interpretability of microbiological predictions. Pouillot and Delignette-Muller [180] developed two R packages, *fitdistrplus* and *mc2d*, to support quantitative microbial risk assessment by modeling uncertainty and variability separately. The *fitdistrplus* package enables statistical distribution fitting and bootstrap-based estimation of parameter uncertainty, whereas *mc2d* supports 2D Monte Carlo simulations that distinguish natural variability from model uncertainty. Their application to *E. coli* O157:H7 risk modeling demonstrated the utility of these tools for transparent and data-driven risk quantification.

Koyama *et al.* [181] extended uncertainty modeling through Bayesian statistical inference to characterize the thermal inactivation of *Salmonella enterica* Typhimurium DT104. By comparing two-step and global Bayesian regression approaches, the authors showed that probabilistic modeling of bacterial inactivation rates produces more reliable and less uncertain estimates than traditional deterministic methods, thereby improving risk-based food-processing design and predictive microbiology.

Building on these probabilistic foundations, Kim *et al.* [182] introduced *ChronoStrain*, a time-aware Bayesian model that tracks low-abundance microbial strains with explicit uncertainty quantification. By incorporating sequence-quality scores and temporal information, the model generates probabilistic abundance trajectories and enables accurate detection of strain-level dynamics, such as post-antibiotic *E. coli* blooms and early-life colonization by *Enterococcus faecalis*. Collectively, these studies underscore the growing importance of uncertainty-aware modeling for enhancing the robustness, transparency, and interpretability of computational microbiology systems.

### Ethical and data governance considerations

Ethical and governance considerations are central to ensuring safe, transparent, and socially responsible approaches to *Salmonella* research and control. Raymond *et al.* [183] emphasize the ethical complexity of conducting controlled human infection model (CHIM) studies for typhoidal *Salmonella* in endemic regions. They argue that, although CHIMs have advanced vaccine development in high-income countries, replicating them in low-resource settings requires context-specific ethical safeguards, including culturally appropriate consent procedures, community engagement, and strong regulatory oversight.

At the policy and industry levels, Chousalkar *et al.* [184] highlight the importance of governance through collaboration among regulators, producers, and researchers in addressing recurring *Salmonella Typhimurium* outbreaks in Australia. Their findings underscore the need for transparent communication, consistent surveillance, and harmonized biosecurity practices to ensure public accountability and food safety.

From a broader One Health perspective, Galán-Relaño *et al.* [185] call for integrated data-governance frameworks linking human, animal, and environmental health in order to manage AMR and cross-sectoral transmission. Their review stresses that coordinated surveillance, predictive modeling, and biosecurity policies are essential for balancing innovation with public protection. Similarly, Wigley [186] reflects on the UK’s long-term governance success in controlling poultry-associated *Salmonella* through structured vaccination, hygiene, and monitoring programs. However, the author cautions that emerging MDR strains challenge existing frameworks and therefore require sustained vigilance, ethical oversight of interventions, and transparent data-sharing practices.

Collectively, these studies demonstrate that ethical responsibility, cross-sector collaboration, and robust data governance are essential for maintaining public trust and ensuring the long-term sustainability of global *Salmonella* control efforts.

### Dataset limitations, overfitting risks, and external validation gaps

A systematic review of representative AMR modeling studies (Table 11) reveals substantial heterogeneity in dataset scale and validation rigor. Sample sizes range from fewer than 100 isolates [141] to reference-scale collections exceeding 10 000 sequences [48], whereas evaluation protocols vary from internal hold-out splits [114, 136, 145, 149] to geographically distinct external cohorts [122, 151]. This variability complicates direct comparison and raises concerns regarding robustness and reproducibility.

**Table 11:** Summary of dataset scale and validation strategies across representative AMR modeling studies. **Evaluation Type** denotes the strongest validation explicitly reported: *Internal* (random hold-out or cross-validation within a single cohort), *External* (evaluation on an independent cohort not used for training), or *Not reported* (the study does not clearly specify the validation protocol). **Cross-site/Species** indicates whether evaluation spans distinct institutions or geographic regions (*cross-site/multi-region*) and whether the analysis is limited to a single species. **Temporal** indicates whether training and evaluation are separated in time. When a paper reports multiple experimental configurations or cohorts, we provide separate rows and label them as *Setting A/B* or *Cohort A/B* to distinguish the dataset or evaluation configuration described in the original study.

Study	Dataset sze	Evaluation type	Cross-site/Species	Temporal
[149] (setting A)	414 isolates	Internal	Cross-site (phylogeny-aware); Single-species	No
[149] (setting B)	1588 isolates (979 genomes)	Not reported	Multi-country; Single-species	Not reported
[151]	339 train; 120 independent	External	Multi-site (China); Single-species	Yes
[122]	5739 isolates	External	Cross-site (India, Israel); Single-species	No
[48]	14 933 reference sequences	External	Not specified	Not reported
[141] (cohort A)	69 isolates	External	Single-site; Single-species	No
[145]	414 isolates	Internal	Single-site; Single-species	No
[144]	3979 genomes	Not reported	Multi-region; Single-species	Not reported
[141] (cohort B)	2379 isolates	Not reported	Single-species	No
[136]	1936 isolates	Internal	Single-species	Not reported
[114]	1936 isolates	Internal	Single-species	Not reported

**Figure 11:**
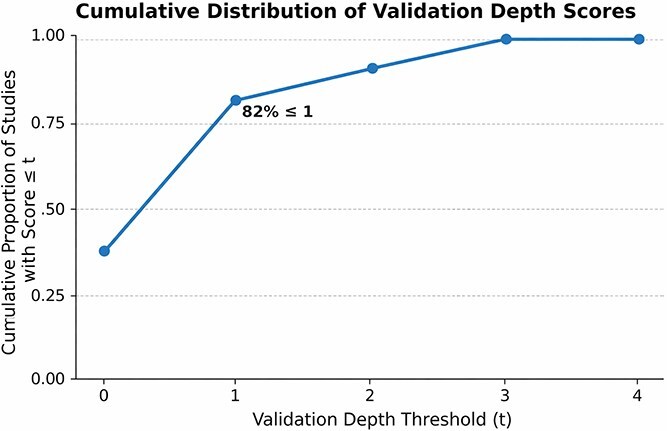
Cumulative distribution of validation depth scores across the reviewed AMR modeling studies. The validation depth score is an additive index reflecting the presence of external cohort evaluation, cross-site validation, temporal validation, and cross-species validation (if reported). The curve highlights concentration at low validation depth, with most studies achieving depth $\leq 1$.

To make the heterogeneity summarized in Table 11 interpretable at a glance, Fig. 11 summarizes validation rigor using an additive validation-depth index comprising external cohort evaluation, cross-site evaluation, temporal validation, and cross-species validation, where applicable. This framing highlights how frequently studies rely on internal validation alone and motivates the risk analyses presented below.

The curve indicates that **82%** of the reviewed studies fall at validation depth $<1$, reflecting a strong reliance on internal evaluation protocols and limited adoption of more rigorous practices such as temporally separated testing or transfer across geographically distinct cohorts. This concentration at low validation depth reinforces the concern that reported performance may not translate under realistic distribution shift.

#### Small sample sizes, class imbalance, and single-cohort evaluation

Dataset size varies by more than two orders of magnitude across studies. For example, [141] report results on only 69 isolates, whereas [48] evaluate 14 933 reference sequences. Several clinically focused studies rely on cohorts containing fewer than 500 isolates (e.g. 414 isolates in [149]; 414 clinical isolates in [145]), which may limit statistical power and increase susceptibility to overfitting. Many studies use internal train–test splits without independent site-held-out cohorts, including [114, 136, 145, 149]. In single-site, single-species collections, such splits may fail to simulate real-world distribution shift, particularly when phylogenetically related isolates appear in both the training and testing sets.

Cross-species validation is rarely performed. Single-species designs are explicitly reported in [114, 136, 141, 144]. Without cross-species evaluation, it remains unclear whether the learned genomic representations generalize beyond organism-specific patterns. In addition, explicit discussion of class imbalance and minority resistance phenotypes is limited across the reviewed studies. Because rare resistance mechanisms may be clinically critical, insufficient representation in the training data may yield inflated macro-averaged metrics that obscure poor minority-class sensitivity.

#### Overfitting risk and limited external validation

True independent external validation remains comparatively uncommon. Among the reviewed studies, only [151] and [122] clearly evaluate models on geographically distinct external cohorts. Even when such cohorts are used, measurable performance degradation is observed. For example, [122] report marked cohort gaps between the India and Israel datasets, including drug-specific discrepancies (e.g. PZA F1 below 0.30 across cohorts and EMB F1 varying from 0.510 to 1.000). Similarly, [149] document macro-F1 reductions of 0.03–0.05 under lineage-aware splitting, with larger drops under certain conditions. These findings suggest that distribution shift can materially affect predictive reliability.

Other studies do not explicitly quantify performance decline under cohort transfer. For example, [48] report a sensitivity gap under identity-threshold stress testing *under stricter sequence-similarity constraints*: at 80% identity, a best-hit baseline yields 0.55 sensitivity, whereas DeepARG achieves 0.99 sensitivity on spike-in evaluation. Although this comparison is not framed as a direct “drop,” it illustrates how evaluation design and similarity thresholds can materially affect apparent robustness. Several studies rely exclusively on internal splits without independent site-held-out validation (e.g. [114, 136, 144]). In these cases, generalization to new hospitals, countries, or emerging resistance profiles remains untested. Collectively, the reviewed evidence indicates that overfitting risk remains nontrivial in AMR modeling, particularly in single-site, single-species studies with moderate sample sizes and no temporal separation between training and evaluation.

### Negative and contradictory findings in AMR modeling

Negative and contradictory findings become more visible when AMR models are evaluated beyond internal splits. A recurring pattern is that apparent gains under in-distribution evaluation may attenuate or even reverse under external validation, reflecting sensitivity to cohort composition, lineage structure, and local epidemiology [122, 149].

First, when external cohorts are considered, performance often degrades relative to within-cohort splits, and many studies either omit geographically distinct validation altogether or do not quantify the magnitude of cross-cohort decline [122, 151]. For example, [122] report marked cross-cohort variability between the India and Israel datasets, with drug-specific discrepancies such as PZA F1 below 0.30 across cohorts and EMB F1 ranging from 0.510 to 1.000. These findings illustrate that in-distribution gains may not transfer under geographic shift.

Second, negative findings are often concentrated in minority resistance phenotypes. Under distribution shift, resistant-class performance may degrade disproportionately even when aggregate metrics appear stable, implying that overall accuracy or macro-averaged scores can mask clinically consequential failure modes. This pattern is consistent with sparse resistant samples, class imbalance, and heterogeneous resistance determinants across settings, and it motivates reporting practices that include class-conditional robustness together with per-drug and per-phenotype performance under external or site-held-out evaluation whenever available.

Third, deployment feasibility can constrain utility even when predictive performance appears favorable. Growth-dependent laboratory processes, fragility in sequencing and bioinformatics pipelines, and stewardship-related implementation overhead introduce time and resource costs that can reduce real-world impact [187–190]. Collectively, these contradictory findings motivate reporting practices that include external validation, class-conditional robustness under shift, and deployment-relevant constraints alongside conventional in-distribution benchmarks.

### Deployment barriers and operational feasibility

Beyond predictive performance, deployment feasibility is shaped by evaluation cost, clinical workflow latency, and the compute and infrastructure requirements needed to operate models in realistic environments. In this section, we synthesize three interlocking classes of barriers that frequently limit the translation of AMR modeling systems into routine practice.

Scalability barriers extend beyond model inference. Many AMR prediction pipelines depend on sequencing throughput, robust quality control, and reproducible bioinformatics workflows; practical failure modes, such as insufficient sample material, extraction failures, and suboptimal sequencing output, can constrain throughput and introduce operational variability [187]. In multi-omics settings, additional scalability constraints arise from modality-specific missingness, batch effects, and harmonization overhead across laboratories and platforms, all of which increase preprocessing burden and can limit cross-site portability. Sustained translation further requires monitoring for epidemiological drift, periodic re-curation of resistance databases, and model updating under changing lineage and drug-use patterns, thereby creating recurring compute, engineering, and governance costs that are rarely accounted for in benchmark-style internal evaluations.

#### Clinical deployment latency: where time is lost in real-world AMR workflows

Clinical deployment latency remains a critical constraint in AMR diagnostics and decision support. Across laboratory and stewardship workflows, the time from specimen collection to therapeutic adjustment is shaped by biological growth requirements, laboratory logistics, analytical processing, and downstream clinical action. Real-world implementation studies demonstrate that improvements in computational or molecular techniques do not automatically translate into proportional reductions in end-to-end clinical turnaround time.

In routine practice, upstream biological constraints often dominate total latency. In [187], the median time to culture positivity for mycobacterial specimens was 20 days, with additional delays attributable to specimen transport and referral to reference laboratories. Although WGS reduced drug-susceptibility reporting time from 12 to 8 days once cultures were available, the time required for species identification increased from 1 to 6 days following implementation. Phenotypic drug susceptibility testing remained substantially slower (median 22 days), and the reported causes of delay included insufficient sample material, DNA extraction failures, suboptimal sequencing output, and bioinformatics processing constraints [187].

Phenotypic AST remains constrained by replication dynamics. As discussed in [188], even accelerated blood-culture-based approaches typically require 10–18 h and may demand substantial hands-on effort, thereby limiting scalability in high-volume laboratories. At the systems level, conventional bloodstream-infection workflows often require 72 h or more from specimen collection to final AST [189].

Latency extends beyond the laboratory. In [190], implementation of a clinical decision-support system reduced the median time to antimicrobial de-escalation from 28.8 to 4.7 h, while measurable pharmacist review time per alert highlighted that human workflow factors introduce additional temporal constraints. Together, these findings motivate end-to-end latency evaluation spanning specimen acquisition through therapeutic modification.

**Key findings: structural drivers of deployment latency**

- Clinical AMR latency is structurally multistage, with dominant delays arising from biological amplification and logistics before computational analysis begins.
- Sequencing and rapid AST technologies redistribute workflow time but rarely eliminate culture-dependent bottlenecks, indicating a biological lower bound on achievable turnaround.
- Meaningful clinical impact requires end-to-end latency reduction spanning specimen acquisition, laboratory processing, model inference, and therapeutic decision-making.

#### L‌LM inference cost and infrastructure barriers in low-resource settings

Evidence from recent healthcare-oriented LLM deployment and systems studies indicates that inference cost and infrastructure feasibility are often first-order determinants of whether an AMR-facing LLM pipeline can be sustained in routine use. These constraints interact with the characteristics of clinical text (e.g. long notes and repeated context), deployment topology (cloud versus edge or fog), and the physical realities of computation (e.g. energy draw, cooling, and hardware availability), thereby shaping both scalability and equity of access.

Cost pressures become concrete when usage is measured in tokens and context length. Klang *et al.* [191] highlight that state-of-the-art models can be expensive at population scale and illustrate this with reported API pricing for GPT-4-32k as of 1 July 2024 ($60 per one million input tokens and $120 per one million output tokens). They further note that higher-capacity, longer-context models often perform better on clinical tasks, thereby creating a utility–cost tension. In this setting, prompt length and context-window limits can become binding constraints when long clinical records must be repeatedly reintroduced for longitudinal reasoning [191].

System architectures that shift execution closer to the point of care are partly motivated by this feasibility gap. Zagar *et al.* [192] describe the expense of cloud-based AI services as a barrier to adoption and motivate edge- or fog-based execution to address privacy, trust, and financial considerations. They emphasize that per-token pricing and limited context windows can make large-scale context injection cost-prohibitive in resource-constrained environments, and that networking and orchestration overhead become additional deployment constraints under decentralized execution [192].

Hardware and energy demand provide an additional lens on inference cost. Samsi *et al.* [193] benchmark LLaMA inference and report that LLaMA 65B inference power draw can range from $\sim$300 W to 1 kW depending on sharding configuration, with energy per generated token reaching several Joules (e.g. $\sim$3–4 J per output token in one setting). They further demonstrate that GPU power capping can reduce total energy consumption while increasing inference time, implying that deployment decisions may be constrained by power budgets and cooling capacity [193].

These cost and compute realities are amplified in low-resource settings. Yang *et al.* [194] discuss remote-care scenarios that rely on lightweight access channels such as SMS via mobile devices and note that resource capacity and technical support may be limited in such environments. They emphasize deployability on constrained devices (e.g. laptops, phones, and Raspberry Pi-class systems) and cost-effective pathways leveraging open-source models [194].

**Key findings: inference cost and infrastructure barriers**

- **Token economics directly constrain clinical scaling:** long-context prompting and repeated inclusion of longitudinal notes translate into per-token cost and create a utility–cost trade-off for high-capacity models [191].
- **Energy and hardware budgets become first-order constraints:** large open-weight models can require substantial power (hundreds of Watts up to $\sim$1 kW) with nontrivial Joules-per-token costs, implying operational limits driven by power availability and cooling capacity [193].
- **Feasible deployment in low-resource settings requires frugal design:** constrained devices, limited technical support, and reliance on lightweight communication channels motivate architectures that reduce compute footprint and bandwidth demand [192, 194].

## Research perspectives and emerging directions

The landscape of AMR research is undergoing a profound transformation driven by the convergence of data-centric modeling, AI, and integrative omics technologies. Beyond incremental advances in genomic prediction, recent work seeks to establish a unified framework that connects molecular mechanisms, environmental dynamics, and clinical translation. This section outlines how computational paradigms in AMR modeling have evolved, identifies the persistent scientific and infrastructural challenges that constrain their impact, and delineates forward-looking trajectories for the next generation of resistance intelligence. Through conceptual taxonomies and analytical synthesis, the discussion integrates three complementary dimensions: (i) current trends and modeling paradigms shaping the field, (ii) the core challenges and emerging solutions spanning data, interpretability, and scalability, and (iii) prospective directions that connect LLMs, FAIR data ecosystems, and agentic AI to transparent and autonomous AMR systems. Together, these perspectives provide a coherent roadmap from foundational analytics to the adaptive, real-world deployment of trustworthy antimicrobial intelligence.

### Current trends in AMR modeling and analysis

Recent advances in AMR modeling reflect a shift from isolated predictive frameworks toward integrated, evidence-grounded, and decision-oriented intelligence systems. Rather than introducing entirely new modeling categories, contemporary research extends the established computational paradigms described in Section Computational paradigms in AMR modeling by strengthening biological grounding, contextual integration, and workflow-level autonomy.

A first major trend is the transition from single-modality predictors to context-aware systems that integrate heterogeneous sources of evidence. Transformer and foundation models increasingly combine sequence embeddings with structural representations and curated biomedical text, enabling resistance mechanisms to be interpreted within functional, pathway-level, and epidemiological contexts. In parallel, retrieval-based architectures explicitly ground predictions in antimicrobial guidelines, curated databases, and the scientific literature, thereby improving traceability and supporting reproducible scientific analysis.

A second emerging direction involves the development of agentic AMR pipelines that operationalize end-to-end reasoning rather than isolated classification. These systems incorporate task decomposition, retrieval-augmented generation, structured tool use, and iterative refinement to support evidence collection, hypothesis generation, and model validation within a unified framework. Increasing attention is also being directed toward uncertainty quantification and selective prediction, particularly in low-data regimes and under distribution shift, in order to ensure reliable decision support in clinical and public health settings.

Finally, contemporary AMR modeling emphasizes evaluation strategies aligned with real-world deployment constraints. Recent studies increasingly assess cross-species generalization, temporal robustness, and performance under incomplete or evolving datasets. Representative evolutionary, generative, and reinforcement-based frameworks that explicitly model resistance emergence and adaptive trajectories are summarized in Table 4. Collectively, these developments indicate a transition from static prediction pipelines toward grounded, uncertainty-aware, and workflow-oriented AMR intelligence capable of supporting continuous discovery in computational microbiology.

**Figure 12:**
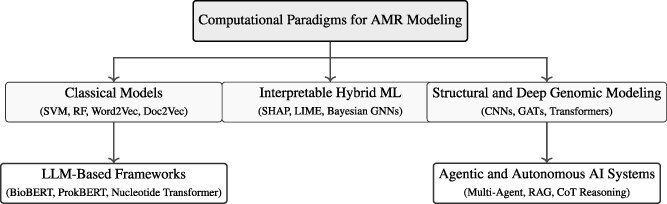
**A conceptual taxonomy of computational paradigms in AMR modeling.** The taxonomy organizes AMR modeling approaches from classical embedding-based and hybrid interpretable machine-learning frameworks to deep genomic architectures and modern LLM-driven agentic AI systems, highlighting the progression toward evidence-grounded, autonomous reasoning in biomedical contexts.

As summarized in Fig. 12, AMR modeling can be organized into a hierarchical taxonomy spanning classical embedding-based approaches, interpretable hybrid models, deep genomic architectures, and emerging LLM-driven systems that emphasize evidence-grounded and autonomous reasoning.

### Key challenges in antimicrobial resistance research

**Figure 13:**
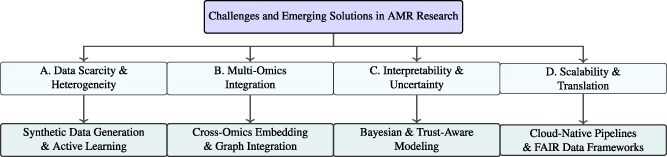
**Challenge–solution taxonomy for AMR research.** A comprehensive taxonomy highlighting the four foundational challenge domains (A–D) that shape the evolution of AMR modeling and their corresponding solution trajectories. The figure emphasizes how innovation across data, representation, interpretability, and deployment converges to enable trustworthy, interpretable, and scalable AMR intelligence.

Despite rapid progress in computational and data-centric approaches, AMR remains one of the most formidable global health threats. Predictive modeling and machine learning have accelerated genomic discovery, yet translation into actionable surveillance or therapy remains constrained by systemic scientific and infrastructural barriers. These challenges are deeply interlinked and extend beyond algorithmic design to issues of data governance, interpretability, and deployment across heterogeneous settings. Figure 13 presents a structured taxonomy of the four central domains that continue to define this frontier: data scarcity and heterogeneity, multi-omics integration, interpretability and uncertainty, and scalability with real-world translation.

#### Data scarcity and heterogeneity

The first obstacle to reliable AMR modeling lies in the uneven distribution, quality, and accessibility of resistance data. Genomic sequencing efforts are concentrated in a small number of well-resourced regions, leaving substantial gaps for emerging pathogens, neglected species, and low-income healthcare systems. This imbalance produces biased training corpora that distort resistance forecasts and weaken cross-domain generalization. Beyond geographic and taxonomic bias, heterogeneity also arises from incompatible experimental designs and inconsistent metadata curation. Variations in antibiotic susceptibility testing, sequencing depth, and annotation pipelines complicate data harmonization. As a result, resistance determinants that appear statistically significant in one dataset may fail to replicate in another, thereby reducing reproducibility and clinical confidence.

To mitigate these disparities, researchers are developing synthetic data-generation, transfer-learning, and active-sampling techniques. Synthetic augmentation can help balance minority classes, whereas active learning prioritizes the most informative isolates for validation. In parallel, standardized metadata schemas and probabilistic record-linkage frameworks are improving interoperability across repositories such as CARD, MEGARes, and ResFinder. The convergence of these efforts is creating a foundation for adaptive data infrastructures that evolve continuously with global surveillance inputs. When combined with FAIR principles of findability, accessibility, interoperability, and reusability, such systems can transform fragmented archives into living resources that sustain reproducible and globally representative AMR analytics.

#### Integration of multi-omics and contextual information

Resistance is a systems-level phenomenon that emerges across molecular, cellular, and ecological scales. Genomic signals alone cannot fully capture the metabolic, regulatory, and environmental contexts that govern antibiotic response. Transcriptomic, proteomic, and metabolomic profiles often reveal complementary dimensions of resistance phenotypes that remain invisible in sequence data. However, integrating these heterogeneous modalities is both technically and conceptually challenging. Each omics layer operates at a different temporal resolution and measurement scale, and naive concatenation risks overwhelming models with noisy, redundant, or conflicting features. Capturing causal relationships among genes, pathways, and phenotypes requires models that can reason across structured and unstructured domains simultaneously.

Recent advances in graph neural networks, attention-based transformers, and cross-omics embedding models are beginning to address this need. These methods map diverse inputs into unified latent spaces in which molecular and environmental dependencies are jointly optimized. When coupled with causal graph learning and domain adaptation, they can infer transferable representations that remain robust under distribution shift. The integration of multi-omics data with epidemiological and ecological context promises a more holistic view of AMR evolution. By embedding clinical metadata, antibiotic-usage patterns, and geographic variables into the learning process, such frameworks can move beyond descriptive correlation toward mechanistic understanding and predictive inference.

#### Interpretability, uncertainty, and trustworthiness

As deep learning becomes increasingly central to AMR prediction, transparency has emerged as a prerequisite for clinical adoption. Black-box algorithms that generate high-confidence outputs without explanatory reasoning raise both ethical and operational concerns. Clinicians must understand not only what a model predicts, but also why it arrives at that conclusion. Interpretability is inseparable from uncertainty quantification. Without calibrated confidence estimates, models may mislead decision makers by expressing false certainty. Overconfident predictions on rare or unseen resistance genes can propagate diagnostic errors, particularly in low-prevalence scenarios where data are sparse.

A new generation of interpretable and uncertainty-aware methods is addressing these limitations. Bayesian neural networks, ensemble calibration, and Monte Carlo dropout enable estimation of epistemic uncertainty, while attention visualization, SHAP, and LIME analyses reveal feature-level contributions to model decisions. Coupling these methods with counterfactual testing further enhances diagnostic accountability. Embedding interpretability into model design, rather than treating it as a purely *post hoc* add-on, creates a foundation for trustworthy AMR intelligence. Systems that expose reasoning pathways and confidence bounds can function as collaborative partners for clinicians, enabling evidence-based decision support rather than opaque prediction.

#### Scalability and real-world translation

Even the most accurate models will remain academic artifacts if they cannot operate efficiently within the realities of healthcare infrastructure. Most AMR analytics pipelines rely on centralized computation and static datasets, thereby limiting responsiveness to newly emerging resistance trends. Deployment across distributed hospitals, laboratories, and national surveillance networks demands scalable architectures that balance performance with accessibility. Technical scalability also intersects with policy and ethical considerations. Data-privacy regulations restrict the movement of clinical isolates, and variations in hardware and network capacity hinder uniform deployment. Federated learning and secure multiparty computation are emerging as viable strategies for training global models without centralized data aggregation.

Cloud-native pipelines, containerized environments, and edge-computing solutions further support decentralized scalability. Such infrastructures allow continuous model retraining, automatic version control, and reproducible analytics under heterogeneous hardware conditions. Combined with FAIR data management, they enable interoperability among research laboratories, public health agencies, and diagnostic facilities. Achieving translation also requires institutional collaboration and transparent validation standards. Harmonized benchmarking protocols and open-access reference datasets can ensure that model claims are testable and comparable. When integrated into national and international health systems, scalable and reproducible AMR frameworks can evolve from isolated experiments into reliable engines of clinical and policy innovation.

These four domains delineate the structural and conceptual frontiers of AMR research. Data scarcity constrains diversity, incomplete integration limits understanding, opaque inference erodes trust, and limited scalability stalls impact. The taxonomy presented in Fig. 13 synthesizes these interdependencies, aligning each limitation with its methodological counterpart. Addressing them collectively is essential for constructing transparent, adaptive, and globally deployable AMR intelligence systems.

### Future outlook and research directions

**Figure 14:**
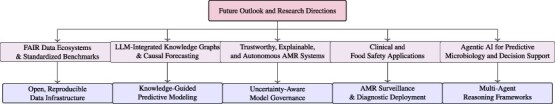
**Taxonomy of future research directions in AMR intelligence.** A hierarchical roadmap illustrating five emerging research pathways: (i) FAIR data ecosystems, (ii) LLM-integrated causal reasoning, (iii) trustworthy autonomous systems, (iv) clinical and food-safety applications, and (v) agentic AI for predictive microbiology and decision support. The progression conveys the transition from foundational data infrastructure to intelligent, applied, and autonomous AMR system.

The next decade of AMR intelligence will be defined by its capacity to merge precision modeling, open data ecosystems, and transparent automation. The scientific objective is no longer limited to predicting resistance, but now extends to enabling continuous and adaptive understanding of microbial evolution across clinical and environmental boundaries. Emerging technologies in large language modeling, graph reasoning, and autonomous systems have the potential to establish a new paradigm in which knowledge extraction, reasoning, and decision support are dynamically integrated. Figure 14 outlines five major trajectories that collectively form a blueprint for this transformation: FAIR data ecosystems, LLM-integrated KGs, trustworthy and explainable AMR systems, clinical and food-safety applications, and agentic AI for predictive microbiology.

#### FAIR data ecosystems and standardized benchmarks

The foundation of next-generation AMR research lies in the construction of open, standardized, and interoperable data ecosystems. Current repositories remain fragmented across disciplines and institutions, with inconsistent metadata standards and incomplete contextual information. Establishing FAIR (Findable, Accessible, Interoperable, and Reusable) data infrastructures is therefore essential for creating a globally connected foundation for transparent model development and reproducibility. These ecosystems must support dynamic updates, ontological consistency, and bidirectional data exchange across genomics, proteomics, and clinical metadata. Beyond data collection, the future also demands community-wide consensus on benchmarking and evaluation. Uniform protocols for dataset curation, feature representation, and task definition will enable reproducible comparisons across models and architectures. Benchmark platforms should reflect the diversity of pathogens, antibiotics, and environmental contexts encountered in real-world surveillance rather than simplified laboratory settings. Open leaderboards and shared evaluation standards will further accelerate progress by establishing transparent performance baselines.

FAIR ecosystems may also benefit from integration with distributed-ledger technologies that ensure traceability and data integrity. Blockchain-based provenance tracking can record the origin, modification, and reuse of AMR datasets, thereby fostering accountability and trust. Coupled with federated access protocols, these mechanisms can protect sensitive patient information while maintaining interoperability across jurisdictions. In the long term, standardized and open data infrastructures will not only facilitate reproducible science but also democratize AMR research. By lowering technical barriers to data access and enabling more equitable participation across global regions, FAIR ecosystems can transform antimicrobial analytics from isolated research efforts into a coordinated, transparent, and continuously evolving scientific enterprise.

#### L‌LM-integrated knowledge graphs and causal forecasting

LLMs offer a transformative opportunity to synthesize and reason over the vast textual and experimental landscape of AMR research. Scientific literature, surveillance reports, and genomic annotations contain implicit causal relationships that remain difficult to access through conventional retrieval systems. Integrating LLMs with structured KGs enables automatic extraction, linking, and inference across multimodal sources, thereby transforming static repositories into dynamic reasoning engines. A crucial future advance will involve aligning LLM embeddings with graph-based representations of molecular and clinical knowledge. This hybrid structure allows models to capture both semantic context and mechanistic causality. By linking molecular entities, drug interactions, and phenotypic outcomes, LLM–graph hybrids can predict resistance trajectories, identify co-selection events, and uncover previously hidden cross-species resistance-transfer patterns.

Causal forecasting represents the next frontier. Instead of merely describing past associations, models will be expected to simulate future resistance scenarios under varying antibiotic-usage patterns or policy interventions. This requires combining counterfactual reasoning with probabilistic causal inference, enabling AI systems to suggest interventions that minimize resistance emergence. Integration with temporal graph neural networks and Bayesian causal-discovery methods can further improve temporal prediction and scenario planning. Ultimately, LLM-integrated causal systems may redefine AMR analytics from a passive diagnostic exercise into an active scientific collaborator. Such models could assist researchers in hypothesis generation, experimental prioritization, and knowledge synthesis, thereby bridging the divide between natural language understanding and molecular evidence-based reasoning.

#### Trustworthy, explainable, and autonomous AMR systems

As AMR intelligence matures, building systems that are trustworthy, interpretable, and capable of autonomous reasoning will become increasingly important. Trustworthiness extends beyond accuracy to encompass transparency, safety, fairness, and reliability under uncertainty. Current deep-learning architectures often provide limited insight into their internal logic, which can result in unanticipated biases or decision inconsistencies. Embedding explainability and uncertainty quantification at every stage of the modeling pipeline will therefore be central to ensuring dependable outcomes in high-stakes applications. The development of explainable architectures will likely involve integrating symbolic reasoning with deep representation learning. Hybrid neuro-symbolic models can combine the flexibility of neural networks with the logical structure of rule-based inference, enabling interpretable decisions supported by scientific rationale. In AMR modeling, this may translate into systems that articulate both statistical predictions and mechanistic hypotheses, thereby providing clinicians with clearer reasoning pathways.

Autonomous functionality will emerge through reinforcement learning and continuous retraining mechanisms that enable models to adapt to new data in real time. Coupled with robust uncertainty estimation, these systems can self-assess their confidence levels and trigger human oversight when reliability thresholds are exceeded. Embedding ethical and safety layers into the decision pipeline is essential to ensure that autonomy remains aligned with human-defined constraints and medical standards. The ultimate vision for trustworthy AMR systems is not to replace human expertise, but to augment it. Transparent and adaptive AI frameworks can function as cognitive amplifiers for scientists and clinicians, allowing them to interrogate, validate, and extend model insights. Establishing this symbiosis between autonomy and accountability will define the next generation of responsible AI in antimicrobial research.

#### Clinical and food safety applications

Translating computational innovation into practical clinical and food-safety applications will mark a decisive shift from predictive research to public-health implementation. Hospitals, diagnostic centers, and agricultural industries require reliable and interpretable systems capable of delivering real-time decision support. AMR surveillance tools must operate seamlessly across these environments, integrating diverse data streams while maintaining accuracy, transparency, and ethical compliance. In clinical settings, next-generation AI systems will increasingly integrate with laboratory information systems and electronic health records to provide rapid resistance predictions. Machine-learning-based antibiograms can be dynamically updated as new isolates are sequenced, thereby reducing diagnostic turnaround times and supporting personalized therapy. Such integration requires standard interfaces, rigorous validation, and regulatory approval processes to ensure compliance with clinical safety standards.

In the domain of food and environmental safety, predictive modeling can help identify contamination sources and trace resistance transmission across the food chain. Monitoring of agricultural antibiotic use and microbial ecology will benefit from real-time data pipelines supported by IoT-enabled biosensors and environmental sequencing platforms. Coupled with predictive analytics, these systems can provide early-warning indicators of resistance hotspots. The convergence of clinical and food-safety intelligence establishes a continuum of AMR surveillance spanning human, animal, and environmental health. This One Health perspective, supported by explainable and interoperable AI systems, can transform global resistance monitoring into a unified decision-support ecosystem. By closing the feedback loop between discovery and deployment, the boundary between research and application becomes more fluid, thereby accelerating both scientific innovation and societal benefit.

#### Agentic AI for predictive microbiology and decision support

The emergence of agentic AI introduces a new paradigm for predictive microbiology. Rather than functioning as passive models, agentic systems operate as autonomous entities capable of reasoning, collaboration, and adaptive problem-solving. In AMR research, multi-agent frameworks can represent distinct expert roles such as data curator, hypothesis generator, experimental designer, and policy advisor, working together to achieve specific research or diagnostic objectives. Technically, agentic systems integrate LLMs with reinforcement learning, retrieval-augmented generation, and dialog-based coordination. Each agent possesses contextual memory and a specialized objective function, allowing agents to share evidence, challenge assumptions, and refine collective outputs. In predictive microbiology, this architecture enables continuous hypothesis testing and scenario evaluation grounded in both biological and environmental evidence.

The next stage of development involves coupling agentic reasoning with multimodal sensing and simulation. Agents could interact simultaneously with genomic, imaging, and clinical data, thereby constructing dynamic causal graphs that capture microbial behavior under environmental and pharmacological perturbations. Integrating simulation feedback into reasoning loops would allow systems to test the impact of interventions before implementation, improving both reliability and interpretability. In the long term, agentic AI may support a self-improving scientific ecosystem in which models generate, evaluate, and validate knowledge collaboratively with human experts. Such systems could serve as the intellectual backbone of future AMR laboratories, enabling continuous learning and cross-domain reasoning. This shift from predictive modeling to collective intelligence marks a defining step toward transparent, evidence-driven discovery in computational microbiology.

#### AMR-specific translation and technical considerations

A substantial portion of the LLM literature cited in this review targets broad biomedical or biological objectives (e.g. clinical text processing, protein and genomics representation learning, or general-purpose biomedical question answering) rather than AMR as a primary endpoint. We therefore distinguish between (i) *direct AMR* applications (e.g. resistance prediction, genotype–phenotype mapping, antibiotic recommendation, and AMR surveillance summarization) and (ii) *transferable* advances from general bio-LLM research that can be adapted to AMR. In particular, improvements in long-context modeling, structured extraction, retrieval-augmented generation, uncertainty-aware generation, and tool-using agents are technically relevant to AMR because AMR workflows often require the integration of heterogeneous evidence streams, including genomics, phenotypes such as MIC and pDST, antimicrobial exposure, and epidemiological context, while also producing auditable outputs for stewardship and surveillance settings. However, AMR translation is not plug-and-play: AMR tasks impose distinct label structures, distribution shifts, and leakage risks that must be addressed explicitly in both problem formulation and evaluation.

Key AMR-specific technical challenges that should be treated as first-order design and evaluation constraints include:

- **Outcome definition mismatch (MIC versus binary resistance):** AMR outcomes are often recorded as continuous MIC values or ordinal susceptibility categories, whereas many machine-learning pipelines reduce labels to binary resistant/susceptible outcomes, potentially discarding clinically meaningful granularity.
- **Severe class imbalance and rare resistance mechanisms:** resistant phenotypes and rare mechanisms may be sparse yet clinically critical, causing instability in minority-class recall and F1 scores and inflating aggregate metrics under imbalance.
- **Site and temporal distribution shift:** resistance prevalence, circulating lineages, and prescribing practices vary across hospitals, regions, and time, making site-held-out and time-held-out evaluation essential for deployment realism.
- **Genomic tokenization and representation constraints:** AMR signals may appear as SNPs/indels, gene presence/absence, plasmid context, or structural variation; naive tokenization can miss biologically meaningful events or inflate sequence length beyond feasible context-window limits.
- **Phylogenetic leakage and nonindependence:** random splits can place closely related isolates in both the training and testing sets, thereby overstating generalization; lineage-aware or cluster-aware splitting is often required.
- **Deployment constraints and auditability:** AMR-facing LLM systems must operate under cost, latency, and infrastructure constraints, and their outputs should be traceable to supporting evidence in order to enable stewardship decisions and validation pathways.

## Conclusion and broader impact

The computational landscape of AMR research has undergone a profound transformation over the past decade, evolving from isolated genomic analyses to integrated, context-aware intelligence systems. This systematic review of 93 studies published between 2016 and 2025 reveals a clear methodological progression: from classical machine learning and feature-engineered classifiers, through deep genomic and structure-informed architectures, to transformer-based foundation models and agentic AI frameworks capable of autonomous reasoning across molecular, clinical, and epidemiological domains. The convergence of multi-omics analytics with LLMs marks a pivotal shift from descriptive pattern recognition toward mechanistic understanding and predictive inference in microbial systems.

### Key findings and synthesis

Our analysis identifies three distinct computational paradigms that collectively define the current state of AMR modeling. **Classical and interpretable machine-learning frameworks**, including Random Forest, XGBoost, and hybrid SHAP-enhanced models, established the foundation for genomic feature attribution and phenotype classification. Although computationally efficient and transparent, these approaches remain constrained by their reliance on handcrafted features and their inability to capture long-range genomic dependencies. **Structural and deep genomic modeling**, integrating AlphaFold-derived protein representations with domain-adaptive attention mechanisms, advanced the field toward mechanistic inference by linking sequence variation to 3D protein conformation and functional consequences. **Transformer-based and applied LLM frameworks**, exemplified by DNABERT, ProkBERT, and Qwen2, represent the current frontier, enabling contextual learning across heterogeneous data modalities and supporting zero-shot generalization in previously unseen resistance contexts.

Beyond individual modeling paradigms, this review highlights four critical integrative directions shaping the future of AMR intelligence. **Embedding-level fusion** establishes unified latent representations across genomic, proteomic, metabolomic, and textual data through projection-based alignment, attention-driven correspondence, and generative synthesis. **KG-enhanced causal reasoning** transforms static biomedical ontologies into dynamic inference engines capable of multi-hop reasoning over gene–pathway–phenotype relationships. **Evolutionary and temporal forecasting models** extend predictive capacity from static resistance detection to dynamic trajectory modeling, capturing mutation dynamics, fitness-landscape evolution, and co-evolutionary host–pathogen interactions. **Agentic AI systems** introduce autonomous reasoning capabilities through multi-agent collaboration, retrieval-augmented generation, and chain-of-thought inference, thereby enabling hypothesis generation, experimental design, and evidence-based validation within FAIR-compliant digital ecosystems.

### Contributions of this review

This work makes several distinctive contributions to the computational microbiology literature. First, we provide a comprehensive synthesis of the intersection between multi-omics integration and LLM applications in AMR research, spanning classical machine learning through emerging agentic AI frameworks. Our PRISMA-compliant methodology ensures systematic coverage and transparent reporting, thereby establishing a reproducible foundation for future meta-analyses. Second, we introduce conceptual taxonomies that organize the fragmented landscape of computational approaches into coherent paradigmatic categories, facilitating cross-study comparison and clarifying methodological evolution. Third, our detailed analysis of preprocessing pipelines, integration strategies, and evaluation frameworks addresses critical gaps in standardization and reproducibility, providing actionable guidance for researchers designing multi-omics studies. Fourth, we explicitly connect technical innovations in AI and deep learning to their biological and clinical implications, thereby bridging the gap between computational methodology and microbiological understanding. Finally, we identify and prioritize future research directions across five domains: FAIR data ecosystems, LLM-integrated KGs, trustworthy autonomous systems, clinical translation, and agentic decision support, thereby providing a strategic roadmap for the next decade of AMR intelligence research.

### Persistent challenges and research priorities

Despite remarkable progress, several fundamental challenges continue to constrain the translation of computational innovation into clinical and public health impact. **Data scarcity and heterogeneity** remain pervasive, with resistance surveillance concentrated in high-income regions and underrepresentation persisting for emerging pathogens, rare resistance mechanisms, and environmental reservoirs. Addressing this imbalance requires coordinated global sequencing initiatives, standardized metadata schemas, and synthetic data-generation strategies to mitigate geographic and taxonomic bias. **Multi-omics integration complexity** presents both technical and conceptual obstacles, as genomic, transcriptomic, proteomic, and metabolomic data operate at different temporal resolutions, measurement scales, and biological hierarchies. Future frameworks must move beyond simple concatenation toward causal graph learning and cross-modal embedding models that capture mechanistic relationships while preserving interpretability.

**Model interpretability and uncertainty quantification** are essential for clinical adoption; however, many state-of-the-art deep-learning models remain difficult to interpret. Integrating Bayesian uncertainty estimation, attention-based explanations, and counterfactual reasoning directly into model architectures, rather than relying on *post hoc* analyses, is critical for developing trustworthy AMR prediction systems. **Scalability and real-world deployment** require federated learning architectures, cloud-native pipelines, and edge-computing solutions that balance computational efficiency with data privacy and regulatory compliance. Establishing harmonized benchmarking protocols, open-access reference datasets, and transparent validation standards will accelerate reproducibility and facilitate meaningful cross-study comparison.

Based on these challenges, we propose five critical research priorities for the next generation of AMR systems: (i) the development of FAIR-compliant, globally representative multi-omics repositories with standardized ontologies and metadata; (ii) the integration of LLMs with structured KGs to enable causal reasoning and counterfactual simulation; (iii) the creation of uncertainty-aware, interpretable AI architectures that provide mechanistic explanations alongside predictions; (iv) the establishment of federated, privacy-preserving frameworks for collaborative model training across institutions and jurisdictions; and (v) rigorous clinical validation studies to assess the real-world performance, safety, and cost-effectiveness of AI-assisted AMR diagnostics.

### Broader impact and vision

The implications of this work extend far beyond computational microbiology, touching on fundamental questions about the future of biomedical AI, data governance, and human–AI collaboration in scientific discovery. The methodological frameworks and integrative paradigms documented here provide a template for addressing complex, multi-scale biological problems across infectious disease research, cancer genomics, and precision medicine. By demonstrating how LLMs can be grounded in structured biological knowledge while maintaining interpretability and causal-reasoning capacity, this review establishes principles applicable to domains that require synthesis of heterogeneous experimental and textual evidence.

From a public health perspective, the transition from reactive surveillance to predictive intelligence has transformative potential for antimicrobial stewardship, outbreak preparedness, and global health equity. Real-time AMR forecasting systems, when deployed responsibly within FAIR and ethically governed frameworks, could enable early detection of emerging resistance hotspots, optimize antibiotic prescribing patterns, and inform evidence-based policy interventions at local, national, and international scales. The integration of clinical, agricultural, and environmental surveillance data within One Health frameworks promises to close critical gaps in understanding resistance transmission across human–animal–ecosystem interfaces.

Looking forward, the convergence of agentic AI, multimodal reasoning, and continuous-learning systems points toward a future in which computational tools evolve from passive predictive models into active scientific collaborators. These systems will not merely forecast resistance, but may also generate testable hypotheses, prioritize experimental designs, simulate intervention outcomes, and iteratively refine their understanding through feedback from laboratory validation and clinical deployment. Realizing this vision will depend on sustained investment in open data infrastructure, interdisciplinary collaboration between microbiologists and AI researchers, and principled governance frameworks that balance innovation with safety, transparency, and equity.

AMR is fundamentally a problem of biological adaptation under selective pressure, a dynamic and evolving challenge that demands equally adaptive and intelligent solutions. The methodological evolution documented in this review, from static feature engineering to autonomous reasoning systems, reflects the field’s growing recognition that understanding and managing resistance require computational frameworks capable of learning, reasoning, and evolving alongside the pathogens they study. By synthesizing current achievements, identifying persistent barriers, and charting forward-looking trajectories, this review provides both a comprehensive assessment of the state of the field and a strategic foundation for the next generation of interpretable, trustworthy, and clinically viable antimicrobial intelligence.

**Key Points**

- Multi-omics integration enables antimicrobial resistance (AMR) prediction beyond single-layer genomic analyses.
- Transformer-based and large language models support context-aware, cross-modal reasoning across genomic, clinical, and epidemiological data.
- Knowledge graphs, evolutionary modeling, and agentic AI systems represent emerging paradigms for predictive microbial intelligence.
- Persistent challenges in data heterogeneity, interpretability, and reproducibility limit clinical translation of AMR models.
- FAIR data infrastructures, uncertainty-aware learning, and retrieval-augmented frameworks provide promising pathways toward deployable AMR intelligence.

## Data Availability

This study did not generate new datasets. All information analyzed in this review was obtained from publicly available literature sources. Details of the literature search strategy and study selection process are provided in accordance with PRISMA 2020 guidelines.
